# Surface Deposition of Dome‐Shaped Metal‐Organic Complexes: A New Approach to the Generation of Single‐Site Catalysts

**DOI:** 10.1002/cplu.202500274

**Published:** 2025-06-28

**Authors:** Küpra Yildiz, Kai Uwe Clausen, Christian Näther, Thomas Strunskus, Felix Tuczek

**Affiliations:** ^1^ Institute of Inorganic Chemistry Christian‐Albrechts‐University of Kiel Max‐Eyth‐Straße 2 24118 Kiel Germany; ^2^ Department of Material Science Christian‐Albrechts‐University of Kiel Kaiserstraße 1 24143 Kiel Germany

**Keywords:** carbonyl ligands, gold, heterogeneous catalysis, molybdenum, surface analysis

## Abstract

A novel approach combining the advantages of heterogeneous with those of homogeneous catalysis is the deposition of metal‐organic complexes on a metallic surface to create well‐defined single‐site catalysts. Dome‐shaped organometallic complexes with weakly binding coligands are well suited for this purpose. With this in mind, a new dithia‐[2.1.1]‐(2,6)‐pyridinophane ligand has been synthesized. The corresponding molybdenum(0) tricarbonyl complex is structurally and spectroscopically characterized in the bulk and in homogeneous solution. Monolayers of this complex are deposited on Au(111) and investigated with the help of surface spectroscopy (infrared reflection absorption spectroscopy, X‐ray photoelectron spectroscopy, and near‐edge X‐ray absorption fine structure). These methods indicate a slightly tilted orientation of the complex on the gold surface, which is confirmed by density functional theory (DFT) calculations. The reactivity of the complex toward dioxygen is evaluated and compared to analogous complexes supported by aza‐ and thiacalix[3](2,6)pyridine ligands.

## Graphical abstract

This study describes the synthesis of a new dithia‐[2.1.1]‐(2,6)‐pyridinophane ligand and its molybdenum(0) tricarbonyl complex. Monolayers of the dome‐shaped complex on Au(111) are investigated by employing surface spectroscopic methods (infrared reflection absorption spectroscopy, X‐ray photoelectron spectroscopy, near‐edge X‐ray absorption fine structure). A slightly tilted orientation is observed and confirmed. The reactivity toward dioxygen is evaluated and compared to analogous systems.

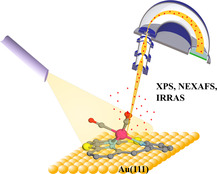

## 1 Introduction

A large number of modern industrial chemical processes and technologies are based on the activation of small molecules by transition metal centers.^[^ ^1^ ^]^ They rely on the chemical transformation of these molecules at well‐defined active sites by coordination to a transition‐metal complex in solution or adsorption to an activated surface (homogeneous or heterogeneous catalysis, respectively).^[^ ^2^, ^3^ ^]^ In order to generate interfaces that combine the advantages of both types of catalysis, the development of well‐defined single‐atom active sites with a high surface density has been at the focus recently, resulting in the generation of single‐site heterogeneous catalysts.^[^ ^4^, ^5^ ^]^ Along these lines, depositing the metal‐organic complexes (MOC) with a single transition metal atom on a substrate can be used to activate small molecules such as H_2_, CO, O_2,_ or N_2_.^[^ ^6^, ^7^, ^8^ ^]^ Coordination of transition metal centers within the plane of the macrocycle (as frequently observed in the case of porphyrins and phthalocyanines),^[^ ^9^ ^]^ generally results in a minimal distance to the surface and, consequently, a pronounced interaction with the latter. Such systems exhibit a reduced reactivity toward gaseous molecules, hindering their coordination to the free coordination site of the transition metal (“surface *trans* effect”).^[^ ^6^, ^7^, ^10^ ^]^

**Figure 1:**
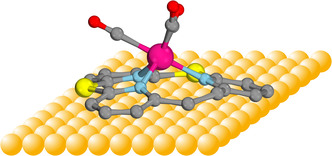
Schematic presentation of surface deposition of a dome‐shaped molybdenum tricarbonyl complex supported by a dithia‐[2.1.1]‐(2,6)‐pyridinophane ligand for the development of a hybrid catalytic system (see text).

In order to overcome the disadvantages associated with planar molecules, dome‐shaped complexes may be employed. These are formed by macrocylic ligands with four donor atoms like calix[4]pyrroles,^[^ ^11^ ^]^ but also more generally by tridentate, cyclic ligands like subporphyrins, triphyrins, calix[3]pyridines, pyridinophanes, or other suitable ligands.^[^ ^12^, ^13^, ^14^, ^15^, ^16^, ^17^, ^18^, ^19^, ^20^, ^21^, ^22^, ^23^ ^]^ By reducing the size of the cavity compared to porphyrins or phthalocyanines, the geometry of these ligands forces the metal center above the ligand plane to adopt a pyramidal rather than a planar coordination geometry, as shown in **Figure** **1**:

The elevation of the coordination center above the ligand plane increases the distance between the transition metal center and the surface, which reduces the surface *trans* effect (while still maintaining some degree of electronic interaction with the surface) and enables the binding or exchange of small molecules at the metal center.^[^ ^12^, ^13^, ^14^, ^15^, ^16^, ^17^, ^18^, ^19^, ^20^, ^21^, ^22^, ^23^ ^]^

Importantly, the fixation of transition metal complexes on metallic surfaces opens a broad range of possibilities for the development of catalytic systems;^[^ ^2^, ^5^ ^]^ e.g., by i) varying the cavity and electronic properties of the ligands that are deposited on the metal surface, thereby altering the distance between the metal center of the complex and the latter, ii) utilizing different transition metals, and iii) employing various metal surfaces.

Recently, our research group has presented two dome‐shaped molybdenum(0) tricarbonyl complexes, supported by two distinct ligands.^[^ ^13^, ^14^, ^15^ ^]^ The complex with *N*‐(*p*‐tolyl)azacalix[3](2,6)‐pyridine as a ligand showed the capability to bind and activate gaseous oxygen (O_2_) in solution as well as deposited on an Au(111) surface, thereby forming a molybdenum(VI) trioxo complex which in turn was capable of catalytic oxygen transfer.^[^ ^13^, ^14^ ^]^ In contrast to the pyridine groups of the *N*‐(*p*‐tolyl)azacalix[3](2,6)‐pyridine ligand, which are linked by tolylamine groups, the thiacalix[3](2,6)pyridine ligand has sulfur atoms at these positions.^[^ ^15^ ^]^ This causes the orientation of the thiacalix[3]‐supported Mo(0) tricarbonyl complex on Au(111) to be exactly parallel to the surface, whereas the azacalix[3]pyridine complex was found to exhibit a slightly tilted orientation due to the additional tolyl groups.^[^ ^13^, ^14^, ^15^ ^]^ Notably, the thiacalix[3]pyridine ligand possesses a larger cavity, leading to a more regular octahedral coordination and a more stable molybdenum(0) carbonyl complex with a better adsorption geometry. Upon deposition, a stronger influence of the gold substrate could be observed in the activation of the carbonyl ligands. These factors, however, resulted in a reduced reactivity toward dioxygen.^[^ ^15^ ^]^

**Scheme 1:**
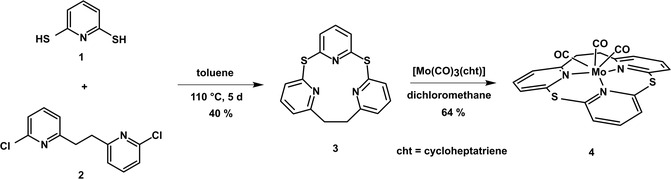
Synthesis of dithia‐[2.1.1]‐(2,6)‐pyridinophane (**3**) (TPn) and the derived molybdenum tricarbonyl complex **4**.

In order to remove this deficiency and investigate whether the alignment to the surface does have an impact on the respective coupling, we developed the novel ligand [2.1.1]‐(2,6)‐pyridinophane that forms a surface‐depositable, dome‐shaped complex. Instead of carbon or oxygen atoms connecting the pyridines, as reported by vedernikov et al. for analogous systems,^[^ ^21^, ^22^, ^23^, ^24^ ^]^ the dithia‐[2.1.1]‐(2,6)‐pyridinophane ligand **3** exhibits bridging sulfur atoms (**Scheme** **1**), which are considered as beneficial regarding the intended surface adsorption (cf Figure 1). The additional ethylene bond serves to expand the ligand's cavity and enhance its flexibility although not allowing the formation of trigonal‐planar complexes.^[^ ^21^, ^22^, ^23^, ^24^, ^25^ ^]^

Herein, the synthesis and characterization of ligand **3** as well as the derived molybdenum(0) tricarbonyl complex **4** are described. **4** is investigated in the solid state, in solution and adsorbed on Au(111) using a range of spectroscopic and analytical methods to determine its electronic and geometric structure. Furthermore, regarding potential applications in catalysis the reactivity of the complex toward dioxygen is evaluated and compared to analogous complexes supported by aza‐ and thiacalix[3]pyridine ligands.

## 2 Results and Discussion

### 2.1 Synthesis and Characterization of Dithia‐[2.1.1]‐(2,6)‐Pyridinophane (3) (TPn) and its Molybdenum Complex 4

**Figure 2:**
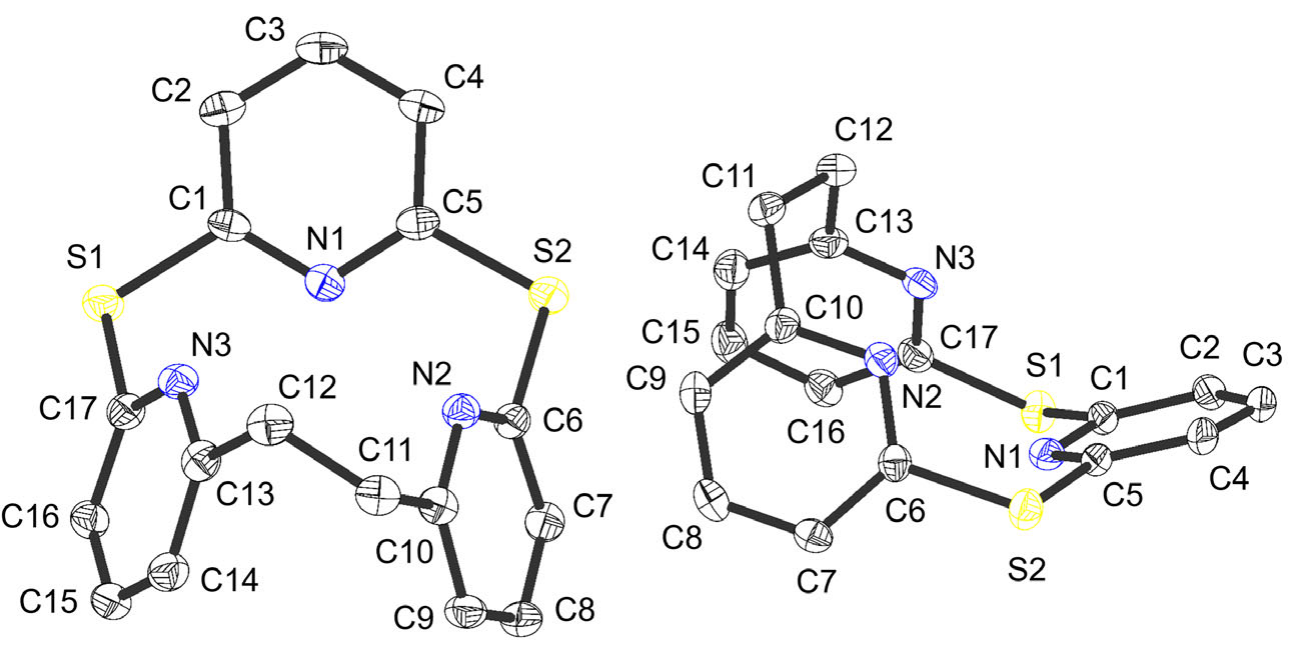
ORTEP plots of dithia‐[2.1.1]‐(2,6)‐pyridinophane (TPn; **3**) with labeling and displacement ellipsoids drawn at the 50% probability level. Hydrogen atoms are omitted for clarity.

The new dithia‐[2.1.1]‐(2,6)‐pyridinophane (TPn) ligand **3** was synthesized with a yield of 40 % by reacting literature‐known 2,6‐dimercaptopyridine (**1**)^[^ ^26^, ^27^ ^]^ and 1,2‐bis(6‐chloropyridin‐2‐yl)‐ethane (**2**)^[^ ^21^ ^]^ in toluene at 110 °C for 5 days (Scheme 1). After column‐chromatographic workup and recrystallization from acetone, crystals of macrocycle **3** suitable for X‐Ray diffraction (XRD) analysis were collected (Table S1, Supporting Information). The structure of **4** is shown in **Figure** **2**.

**3** adopts an almost triangular shape, with angles of the ethylene bridge being 117.7° (N2‐C10‐C11) and 115.8° (N3‐C13‐C12). Due to the electrostatic repulsion between the lone pairs of the pyridine nitrogen atoms, the pyridine ring between the thioether bridges is flipped downwards with respect to the two other pyridines (cf. Figure 2 right). This peculiarity is also observed for the free azacalix[3]pyridine^[^ ^28^ ^]^ and thiacalix[3]‐pyridine^[^ ^29^, ^30^ ^]^ ligands. The average C—S bonds length of 1.787 Å as well as the C—S—C angles for the dithia‐[2.1.1]‐(2,6)‐pyridinophane ligand **3** (99.9° for C1—S1—C17 and 102.5° for C5—S2—C6) are in the same range compared to the thiacalix[3]pyridine ligand (1.780 Å and 101.9°, respectively).^[^ ^29^, ^30^ ^]^

**Figure 3:**
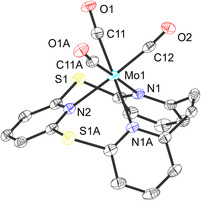
ORTEP plot of [Mo(CO)_3_(TPn)] (**4**) with labeling and displacement ellipsoids drawn at the 50% probability level. Hydrogen atoms and the dichloromethane are omitted for clarity. Symmetry code for the generation of equivalent atoms: A: *x*, ‐*y* + 3/2, *z*.

The complex [Mo(CO)_3_(TPn)] (**4**) was obtained as a red powder with a yield of 64 % by mixing TPn (**3)** with 1 eq of [Mo(CO)_3_(cht)] dissolved in dichloromethane under nitrogen atmosphere (Scheme 1). Crystals of **4**·CH_2_Cl_2_ suitable for single‐crystal XRD (SC‐XRD) were obtained from a dichloromethane solution by slow evaporation under argon atmosphere (Table S1, Supporting Information). The structure of **4** is shown in **Figure** **3**; selected bond lengths and angles are listed in **Table** **1**.

**Table 1:** Selected bond lengths [Å] and angles [°] of [Mo(CO)_3_(TPn)] (4).

Mo(1)‐C(11)	1.940(3)	N(1’)‐Mo(1)‐C(11)	178.55(11)
Mo(1)‐C(11’)	1.940(3)	N(2)‐Mo(1)‐C(12)	177.67(15)
Mo(1)‐C(12)	1.935(5)	N(1)‐Mo(1)‐C(11)	96.37(11)
Mo(1)‐N(1)	2.332(2)	N(1)‐Mo(1)‐C(12)	99.78(11)
Mo(1)‐N(1’)	2.332(2)	N(1’)‐Mo(1)‐C(11’)	96.37(11)
Mo(1)‐N(2)	2.332(4)	N(1’)‐Mo(1)‐C(12)	99.78(11)
C(11)‐O(1)	1.168(4)	N(2)‐Mo(1)‐C(11)	96.70(11)
C(11’)‐O(1’)	1.168(4)	N(2)‐Mo(1)‐C(11’)	96.70(11)
C(12)‐O(2)	1.168(6)	C(11)‐Mo(1)‐C(11’)	84.29(18)
		C(11)‐Mo(1)‐C(12)	81.58(13)
N(1)‐Mo(1)‐N(1’)	82.94(12)	C(11’)‐Mo(1)‐C(12)	81.58(13)
N(1)‐Mo(1)‐N(2)	81.94(9)	Mo(1)‐C(11)‐O(1)	173.9(3)
N(1’)‐Mo(1)‐N(2)	81.94(9)	Mo(1)‐C(11’)‐O(1’)	173.9(3)
N(1)‐Mo(1)‐C(11’)	178.55(11)	Mo(1)‐C(12)‐O(2)	174.5(4)

The SC‐XRD analysis reveals that the Mo(0) center of **4** is octahedrally coordinated by three carbonyl ligands and the TPn ligand **3**. The complex **4** has an average Mo—N bond length of 2.332 Å, an average Mo—C bond length of 1.938 Å, and an average CO bond length of 1.168 Å. This is consistent with values for other molybdenum tricarbonyl complexes supported by cyclic tridentate *N*‐donor ligands.^[^ ^31^, ^32^, ^33^, ^34^, ^35^, ^36^ ^]^ Both the N—Mo—N angles of the Mo(TPn) unit and the C—Mo—C angles of the Mo(CO)_3_ unit are smaller than 90°, corresponding to a trigonal elongation of the coordination sphere. The N—Mo—C angles are ≈180° (average value of 178.26°). In agreement with the *C*_s_ symmetry of the complex, slightly different C—Mo—C and, respectively, N—Mo—N angles are observed. The C(11)—Mo(1)—C(12) and C(11’)—Mo(1)—C(12) angles, e.g., have a value of 81.58° whereas the C(11)—Mo(1)—C(11’) angle (84.29°) is 2.71° larger. The N—Mo—N angles are consistent with those of other molybdenum(0) carbonyl complexes coordinated by tridentate *N*‐donor ligands,^[^ ^13^, ^15^, ^31^, ^32^, ^33^, ^34^ ^]^ although, with an angle of 82.27°, the average N—Mo—N angle in [Mo(CO)_3_(TPn)] (**4**) is at the upper end of the values observed for such systems so far.^[^ ^13^, ^15^, ^34^, ^35^, ^36^ ^]^ In particular, the N(1)—Mo(1)—N(1’) angle, including the ethylene bridge, is by 1° larger than the N(1)—Mo(1)—N(2) angles comprising sulfur bridges. This reflects the ring expansion of the tridentate macrocycle due to the ethylene bridge. In contrast, the azacalix[3]pyridine ligand has a smaller cavity, leading to significantly smaller angles and bond lengths in the derived Mo(0) tricarbonyl complex,^[^ ^13^ ^]^ whereas the angles and bond lengths for thiacalix[3]pyridine ligand only differ slightly.^[^ ^15^ ^]^

A more thorough look at the crystal structure of **4** reveals an additional noteworthy feature. When an axis is drawn from the carbonyl C via the molybdenum to the *trans*‐N atom, it becomes evident that the plane of the pyridine rings either deviates upwards or downwards relative to this axis. The sulfur‐bridged pyridine is tilted upward by roughly 24°, whilst the remaining two pyridines are tilted downward by around 17° (see Figure S11 and S12, Supporting Information). This structural feature is of particular importance for the near‐edge X‐ray absorption fine structure (NEXAFS) and infrared reflection absorption spectroscopy (IRRAS) measurements (see below).

[Mo(CO)_3_(TPn)] (**4**) was also spectroscopically characterized in the bulk and in solution. High‐resolution NMR spectra can be found in (Figure S5 and S6, Supporting Information). The assignment of all resonances to the respective carbon atoms was made possible by employing 2D correlation NMR techniques and distortionless enhancement by polarization transfer spectroscopy (DEPT‐135‐spectroscopy). However, in this instance, due to the low solubility of complex **4**, the signal of the CO ligands could not be observed in the ^13^C‐NMR spectrum.

**Figure 4:**
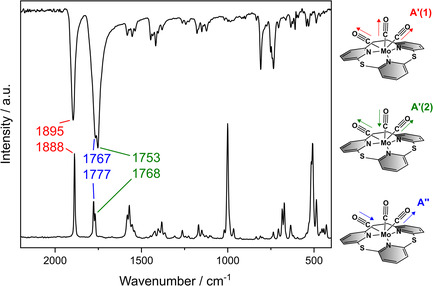
IR (top) and Raman (bottom) spectra of [Mo(CO)_3_(TPn)] (**4**) with the illustration of the observed CO vibration (right).

Solid‐state IR and Raman spectra of **4** are shown in **Figure** **4**. Importantly, they provide information regarding the activation of the carbonyl ligands coordinated to the Mo(0) center. In agreement with the *C*_s_ symmetry of the complex (see above), the presence of three carbonyl bands is anticipated in the IR and Raman spectra. While all modes are IR‐ and Raman‐allowed, their relative intensities differ in the corresponding spectra. The symmetric A’(1) vibration is found at 1895 cm^−1^ in the IR and at 1888 cm^−1^ in the Raman spectrum (Figure 4, red). In this case, all three carbonyl ligands oscillate in phase, as illustrated in Figure 4.

Due to the symmetry reduction of *C*_3v_ to *C*_s_, the E vibration splits into A'(2) and A” vibrations. The A'(2) vibration is an out‐of‐phase combination of a symmetric stretching motion of the “equatorial” CO ligands and a stretch of the “axial” CO ligand (whereby “equatorial” refers to the plane containing the ethylene‐bridged pyridines and the carbonyl ligands *trans* to it). This vibration is found at 1767 cm^−1^ in the IR and 1777 cm^−1^ in the Raman spectrum (Figure 4, blue). Similarly, the A” band (IR 1753 cm^−1^, Raman 1768 cm^−1^, Figure 4, green) corresponds to the antisymmetric stretching motion of the “equatorial” CO ligands with the “axial” CO ligand being at rest. Comparison of CO stretching frequencies of the carbonyl ligands with ν(CO) reported for free carbon monoxide (2143 cm^−1^)^[^ ^37^ ^]^ reveals a significant activation of the CO bond. This is in agreement with the results of the SC‐XRD analysis (cf. above) and with other studies of molybdenum(0) carbonyl complexes reported in the literature.^[^ ^13^, ^15^, ^31^, ^32^, ^33^, ^34^, ^35^, ^36^ ^]^

### 2.2 Deposition of 4 on Au(111) and Investigation with Surface Spectroscopic Methods

Complex **4** was deposited on Au(111) by immersing the gold substrate into a solution of **4** (see Experimental Section). The resulting monolayers were investigated by polarization‐modulation infrared absorption spectroscopy (PM‐IRRAS), X‐ray photoelectron spectroscopy (XPS), and near‐edge X‐ray absorption fine structure (NEXAFS).

#### 2.2.1 IRRAS

**Figure 5:**
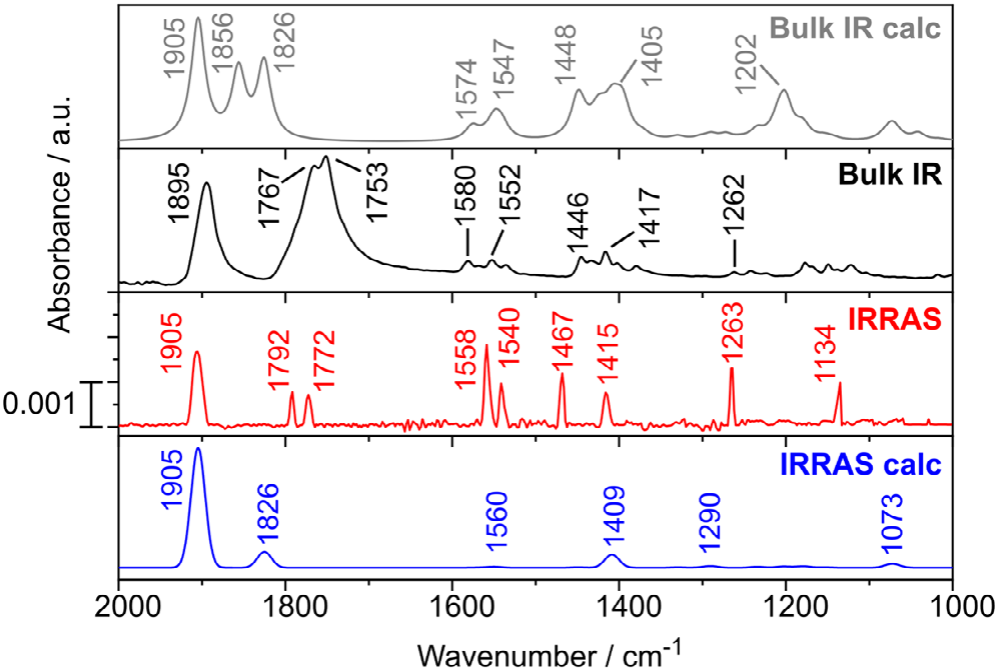
Calculated and measured vibrational spectra of [Mo(CO)_3_(TPn)] (**4**) in bulk material and adsorbed on Au(111) surfaces.

The PM‐IRRA spectrum of [Mo(CO)_3_(TPn)] (**4**) absorbed on Au(111) is shown in **Figure** **5** along with the bulk IR spectrum and calculated IR and IRRA spectra. The range of 10^−3^ absorbance units is indicative of the formation of a monolayer on Au(111).^[^ ^13^, ^15^, ^38^, ^39^, ^40^ ^]^ Differences in relative intensities of the bands between the bulk IR spectrum and the IRRA spectrum provide information on the orientation of the molecules on the surface, based on the surface selection rule of IRRA spectroscopy.^[^ ^41^ ^]^ In simulating the IRRA spectrum, complex **4** was aligned so that the plane through the three pyridine nitrogen atoms is located parallel to the surface (xy plane). It is important to note that the calculation of the IRRA spectrum was conducted without taking the gold surface and its influence at an atomic level into account. The calculated bulk and IRRA spectra largely align with the measured IR and IRRA spectra (Figure 5). Accordingly, the observed bands could be assigned to specific vibrational modes (Table S2, Supporting Information).

The most significant bands in the bulk IR spectrum are the stretching vibrations of the carbonyl ligands, some of which can also be observed with IRRAS. The symmetric A'(1) mode is located at 1905 cm^−1^ in the IRRA spectrum. Due to the *C*_s_ symmetry, the E vibration splits into the A’(2) and A’’ vibrations (see above). Both vibrations can also be observed with smaller relative intensities at 1792 cm^−1^ (A’’) and 1772 cm^−1^ (A’(2)) in the IRRA spectrum. The calculation of the IRRA spectrum indicates that only the A’(2) vibrational mode should be visible (with low intensity) under the assumption that the plane through the three pyridine nitrogen atoms is parallel to the surface. However, the observation of all three carbonyl vibrations reveals that the central Mo(CO)_3_N_3_ core of complex **4** must be slightly tilted with respect to the surface normal. As apparent from the crystal structure, the sulfur‐bridged pyridine is less tilted with respect to a plane that is formed by the three nitrogen atoms than the other two pyridines linked by the ethylene bridge. As a consequence, the molecule is tilted toward the side of the sulfur‐bridged pyridine. Therefore, the transition dipole moment (TDM) of the A” vibration gets a component along the z‐axis and can therefore also be observed with low intensity in the IRRA spectrum according to the surface selection rule.

Most of the ligand signals in the measured bulk IR can also be observed in the measured IRRA spectrum (bulk IR: 1580, 1552; 1446, 1417, 1262 cm^−1^; IRRAS: 1558, 1540; 1447, 1415, 1263 cm^−1^). However, they differ in intensity, which can also be explained by the orientation of the complex **4** on the surface and the selection rules of IRRA spectroscopy. Only three of the four IRRA calculated vibrational modes of the ligand **3** can be seen in the measured IRRA spectrum (1558, 1415, and 1263 cm^−1^). The former two belong to the C—C stretching modes of the pyridine rings, while the latter corresponds to the deformation vibration of the pyridine ligand. The latter also differs by a large amount from the calculated intensity at 1290 cm^−1^. In addition, another C=C stretching mode at 1540 cm^−1^ can be observed, whereas the C—H bending mode at 1073 cm^−1^ is missing. These observations can be traced back to the fact that the influence of the gold substrate was not taken into account in the IRRAS calculation.

A noteworthy observation when comparing the IRRA to the bulk IR spectrum is the shift of the carbonyl stretching vibrations to higher wavenumbers. This phenomenon is attributed to the electronic influence of the metallic substrate, as electron density is transferred from the Mo(0) complex to the surface (“static (de)activation”). This is in contrast to the process known as “dynamic activation”, in which the flow of charge occurs from the metal center into the π* orbitals of the carbonyl ligands during the extension of the C—O bonds. This counteracts the initial effect and only applies to the fully symmetric A’(1) vibration, where all CO ligands oscillate in phase.^[^ ^13^, ^15^, ^38^, ^39^ ^]^ The symmetric A'(1) vibration shifts by 10 cm^−1^, and the A’’ and A'(2) vibrations shift to higher wavenumbers by 25 and 19 cm^−1^, respectively. Thus, a deactivating shift of the carbonyl vibration of complex **4** can be observed.

A comparison of the A'(1) vibration of complex **4** with those of azacalix[3]pyridine (6 cm^−1^) and thiacalix[3]pyridine (20 cm^−1^) places it between these structures. However, the A'(2) and A’’ vibrations exhibit a stronger shift compared to the azacalix[3]pyridine system (8 and 9 cm^−1^).^[^ ^15^ ^]^ This could be attributed to the more significant influence of the gold substrate in comparison to that observed in previously studied complexes. The enhanced interaction with the surface compared to the azacalix[3]pyridine system results in a stronger coupling, leading to a significantly stronger deactivating effect.

#### 2.2.2 XPS

XPS was used to determine whether [Mo(CO)_3_(TPn)] (**4**) was deposited on the gold surface (Au(111)) intact and without impurities. A survey spectrum as well as high‐resolution spectra of the different regions were acquired in order to assign the binding energies of the different species contributing to the N 1s, C 1s, S 2p, and Mo 3d spectra with acquisition times of up to one hour.

**Figure 6:**
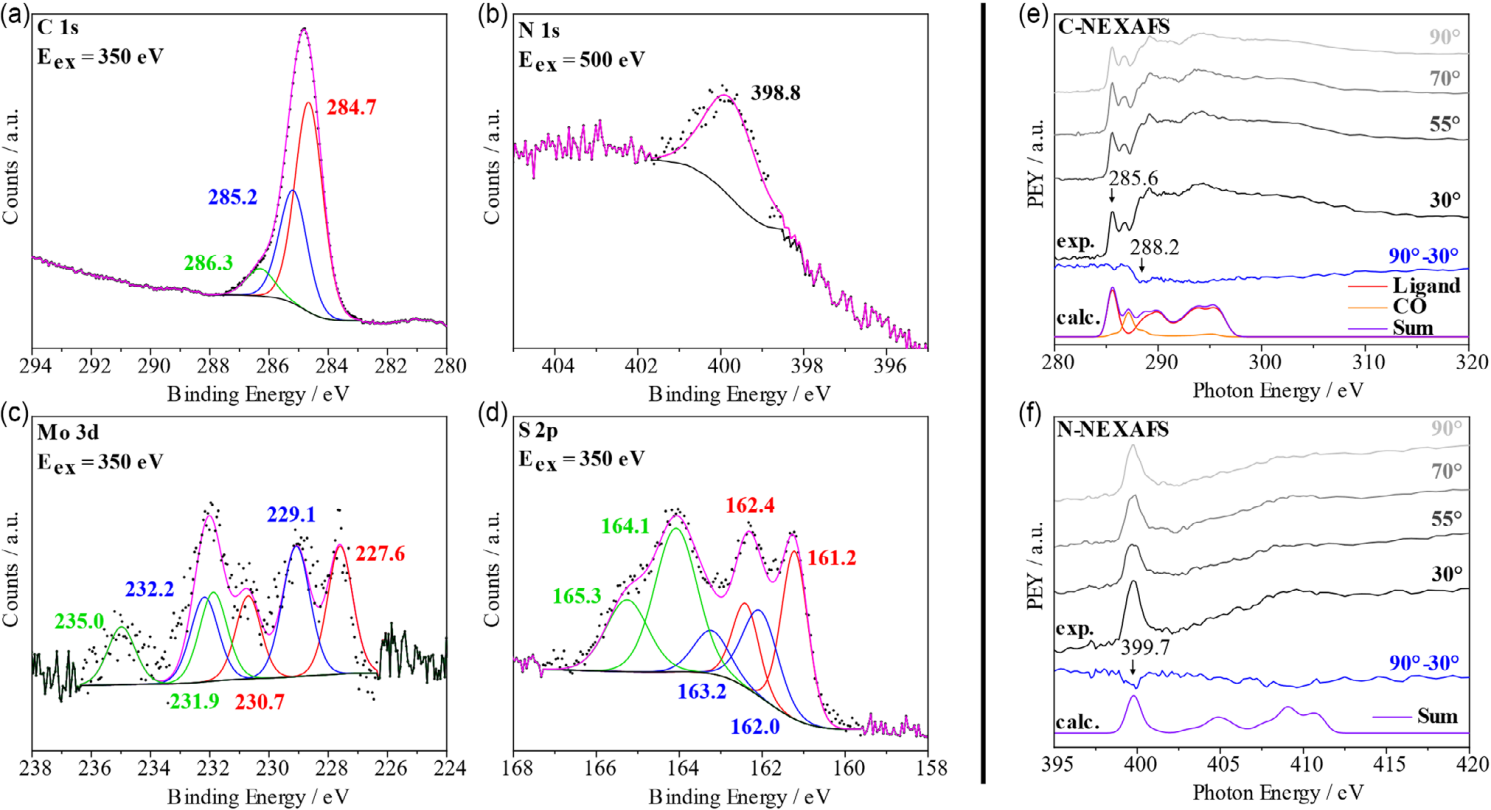
XP spectra of a monolayer of [Mo(CO)_3_(TPn)] (**4**) on Au(111) of C 1s a), N 1s b), Mo 3d c), and S 2p d) and normalized NEXAFS spectra of a monolayer of [Mo(CO)_3_(TPn)] (**4**) on Au(111) at different angles (30°, 55°, 70°, 90°) and the calculated NEXAFS data (shifted by +10.9 eV for C, +12.8 eV for N) with the contributions of the different subunits added together over all angles: e) C K‐edge spectrum; f) N K‐edge spectrum.

The XP spectra of complex **4** are collected in **Figure** **6**. In the survey spectrum (Figure S16, Supporting Information), the Au 4f signal at 84.0 and 87.5 eV appears predominantly. Furthermore, the Au 4d signals at 335.0 and 352.5 eV, and the C 1s peak at 285 eV are clearly visible. The intensity ratio of the C 1s signal to the Au 4d signals indicates the presence of a monolayer of **4** on the gold surface. The N 1s (399 eV), the Mo 3d (228 eV), and the S 2p signal at 161 eV were identified as well.^[^ ^13^, ^15^, ^38^, ^39^, ^40^, ^42^ ^]^

The C 1s spectrum (Figure 6a) can be deconvoluted into three signals, with the most intense signal at 284.7 eV (red) representing carbon atoms linked to each other. The less intense species at 285.2 eV (blue) can be identified with carbon atoms bound to heteroatoms such as nitrogen and sulfur. The third signal at 286.3 eV (green) corresponds to carbon atoms that are attached to oxygen.^[^ ^39^, ^43^, ^44^ ^]^ The intensity ratio of the three species was measured to be 60:32:8, which is not consistent with the theoretical ratio of 68:20:12 derived from the molecular composition of complex **4**. The complex thus appears to have less than three CO ligands, which would account for the lower ratios of the carbon–carbon and carbon–oxygen bonds. This could be attributed to the decomposition of the complex **4** due to the effects of the synchrotron radiation. This assumption is further supported by the Mo 3d spectrum (see below).

In the N 1s XP spectrum (Figure 6b), one signal is observed at 398.8 eV, as expected. This species is attributed to the pyridine nitrogen atoms.^[^ ^13^, ^15^, ^38^, ^43^ ^]^

The Mo 3d XP spectrum (Figure 6c) contains three doublets with a characteristic splitting of 3.1 eV and an intensity ratio of 3:2.^[^ ^13^, ^15^, ^38^, ^39^ ^]^ The first species at 227.6/230.7 eV (red) corresponds to molybdenum(0),^[^ ^13^, ^15^, ^45^, ^46^, ^47^ ^]^ whereas the second doublet at 229.1/232.2 eV (blue) and the third doublet at 231.9/235.0 eV (green) are attributed to molybdenum species in oxidation states of +4 and +6, respectively.^[^ ^45^, ^46^, ^47^, ^48^ ^]^ The intensity ratio of Mo(0):Mo(IV):Mo(VI) is 35:39:26, indicating partial conversion of molybdenum from the oxidation state of 0 to +4 and +6. Notably, this observation is consistent with the C 1s spectrum, indicating the dissociation of carbonyl ligands of [Mo(CO)_3_(TPn)] (see above). The oxidation is attributed to the high‐energy synchrotron radiation along with traces of oxygen, a finding that has also been observed in our previous studies.^[^ ^13^, ^15^ ^]^ While full conversion of the molybdenum center up to the oxidation state of +6 was observed in the azacalix[3]pyridine system,^[^ ^13^ ^]^ oxidation of the thiacalix[3]pyridine complex under the conditions of XPS was found to only lead to a charge state of +4.^[^ ^15^ ^]^

From these observations, it was concluded that the larger ring size of the thiacalix[3]pyridine ligand (with respect to its azacalix[3]pyridine counterpart) leads to a more stable Mo(0) complex, which in turn reduces its reactivity toward oxygen. The dithia‐[2.1.1]‐(2,6)‐pyridinophane (TPn) ligand possesses an even larger cavity than thiacalix[3]pyridine, but exhibits a flexible ethylene bridge. This flexibility obviously renders the TPn complex more reactive toward O_2_ than its thiacalix[3]pyridine‐supported counterpart. More information on this issue is provided below.

The S 2p spectrum, finally, contains three species with the characteristic spin‐orbit splitting of 1.18 eV, and an intensity ratio of 2:1 (Figure 6d).^[^ ^49^, ^50^, ^51^, ^52^ ^]^ The main signal at 163.9 and 165.1 eV (green) can be assigned to the two bridging sulfur atoms of **4**.^[^ ^49^, ^53^ ^]^ Based on the geometry observed in Figure 3, the two lone pairs of the sp^3^‐hybridized sulfur atoms should, in theory, not interact with the gold surface. Nonetheless, the thioether bridge has the capacity to flip downwards, enabling one of the sulfur lone pairs to bind to the surface. The doublet at 162.3/163.5 eV (blue) is attributed to the characteristic Au—S bond.^[^ ^49^, ^50^, ^51^ ^]^ The third species, observed at 161.2/162.4 eV (red) is typically attributed to adsorbed sulfur atoms or organothiol species on gold surfaces derived from thiol‐derived self‐assembled monolayers (SAMs).^[^ ^51^, ^54^ ^]^ The first source of this additional species could be due to the decomposition of the molecules, forming chemisorbed sulfur. Alternatively, the presence of another sulfur species may result from an impurity. However, no evidence for these possibilities is provided by other spectroscopic and analytical methods. This additional sulfur signal was also observed in the XP spectra of Mo(0) tricarbonyl complex with thiacalix[3]pyridine as a ligand.^[^ ^15^ ^]^

In conclusion, also on the basis of XP spectra of a thick layer of **4** (Supporting Information Chapter 4), the XP spectra confirm that [Mo(CO)_3_(TPn)] (**4**) is deposited intact and with a high purity on gold, forming a monolayer. However, the presence of high‐energy radiation appears to induce dissociation of the carbonyl ligands from the metal center, along with partial oxidation of the molybdenum centers. Nevertheless, the ligand maintains its integrity and remains bound to the molybdenum center.

#### 2.2.3 NEXAFS

NEXAFS spectra were measured at the carbon and nitrogen K‐edges for a monolayer of [Mo(CO)_3_(TPn)] (**4**) on Au(111) at different angles (Figure 6e,f). This method was employed to determine the orientation of the complex **4** adsorbed on the surface more precisely.

In Figure 6e the C K‐edge spectrum contains three major π* resonances which can also be found in the DFT‐calculated spectrum (Figure 6e, purple trace). Two π* resonances at 285.6 and 288.2 eV are assigned to a C 1s to π* transition of the pyridine units, whereas the third π* transition signal at 286.8 eV belongs to the CO ligands.^[^ ^13^, ^15^, ^38^ ^]^ The three signals exhibit only a small angular dependence, as evidenced by the intensity difference between 30° and 90°, whereby the distinction between the resonance of the CO ligands is only barely visible. The same observation is also evident in the N K‐edge spectrum in Figure 6f, which displays a single π* resonance at 399.7 eV. The assignment of this resonance to the N 1s to lowest unoccupied molecular orbital (LUMO) transition of the pyridine units is supported by DFT calculations and previous studies.^[^ ^13^, ^15^, ^38^ ^]^ In comparison to the C‐NEXAFS spectrum, the intensity of this transition also exhibits only a slight decrease with increasing angle of incidence.

Due to the different upward and downward tilts, the pyridine rings of **4** exhibit significantly distinct orientations in space and also with respect to the gold surface (see above). In combination with a slightly tilted adsorption geometry of molecule **4** (see IRRAS), a decreasing intensity of the π* resonances is expected for the ethylene‐bridged pyridines with increasing angle of incidence in both spectra (C‐ and N‐NEXAFS). On the contrary, the sulfur‐bridged pyridine exhibits the opposite angular dependence regarding the intensity of the transition from C 1s (or N 1s) to π*. This results in a cancelation of the angular dependences of the resonant transitions due to the two different pyridine ring orientations. Notably, the 2:1 ratio of the pyridine rings in the molybdenum complex **4** causes a slight overall intensity decrease from 30° to 90°, in line with the observed decrease in intensity and the minor angular dependence observed in the C‐ and N‐NEXAFS spectra. Taken together, these findings thus confirm the tilted adsorption geometry of complex **4** on the gold surface.

### 2.3 Reactivity of 4 Toward Dioxygen (O_2_)

The preceding spectroscopic investigations of the molybdenum complex **4**, in particular the XPS data, indicate a higher reactivity of this complex toward dioxygen than observed for the analogous complex supported by the thiacalix[3]pyridine ligand.^[^ ^15^ ^]^ Thus, we speculated that a targeted oxygenation of **4** might be possible. Notably, the reactivities of molybdenum(0) tricarbonyl complexes supported by azacalix[3]pyridine and thiacalix[3]pyridine ligands toward dioxygen in solution were found to be different: while the former, upon exposure to molecular oxygen, showed conversion to a Mo(VI) trioxo complex within one hour,^[^ ^14^ ^]^ the same reaction with the latter resulted in loss of the metal center.^[^ ^15^ ^]^ Given the similarity to both of these ligands to the dithia‐[2.1.1]‐(2,6)‐pyridinophane ligand **3**, it appeared of interest to also explore the reactivity of the derived molybdenum carbonyl complex **4,** exhibiting a larger, but more flexible cavity than its predecessors, toward O_2_ in homogeneous solution. To this end, **4** was dissolved in dichloromethane under an inert atmosphere and exposed to oxygen. Over the course of several hours, the solution underwent a color change from an intense red to a dark brown. After one day, the solution had turned colorless, accompanied by the formation of a greenish‐gray precipitate. This result indicates that, similar to the thiacalix[3]pyridine system,^[^ ^15^ ^]^ an oxidation followed by a demetalation process might have occurred. The free ligand **3** was spectroscopically identified in solution (Figure S21, Supporting Information) and the gray‐colored precipitate was further investigated by X‐ray diffraction (Figure S22, Supporting Information) and IR spectroscopy (Figure S23, Supporting Information). However, the composition of the precipitate could not be determined.

**Figure 7:**
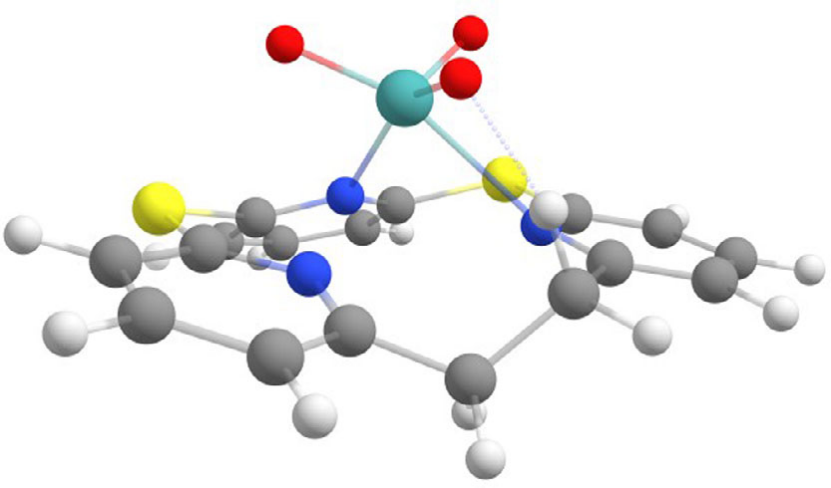
Structure of a hypothetical [MoO_3_(TPn)] (**5**) complex predicted by DFT calculation.

In order to account for the described results, DFT calculations were performed in analogy to the previously reported ligand systems.^[^ ^14^, ^15^ ^]^ The structure of the hypothetical molybdenum(VI) trioxo complex [MoO_3_(TPn)] (**5**) supported by the dithia‐[2.1.1]‐(2,6)‐pyridinophane ligand **3** was successfully optimized (**Figure** **7**). Complex **5** exhibits an O—Mo—O angle of 106.6°, which falls within the typical range for MoO_3_ complexes (105°–108°).^[^ ^15^, ^33^, ^55^ ^]^ Moreover, a hydrogen bond forms between one oxo ligand and the ethylene bridge. In comparison to the parent Mo(0) carbonyl complex **4,** the Mo—N bond length increases drastically to an average of 2.464 Å (cf. **Table** **2**), and a bond between Mo and one of the coordinating pyridine nitrogen atoms breaks. This results in a distorted trigonal bipyramidal coordination sphere. Moreover, the average N—Mo—N bond angle increases by up to 5°. With 77.32°, the N—Mo—N angle is greater than those of the azacalix[3]pyridine and thiacalix[3]pyridine complexes (cf Table 2), which is again due to the increased cavity size of the TPn ligand **3**.^[^ ^14^, ^15^ ^]^

**Table 2:** Average of selected bond lengths [Å] and angles [°] of [Mo(CO)_3_(TPn)] (4), hypothetical [MoO_3_(TPn)] (5) complex and for previously investigated complexes (ACP = azacalix[3]pyridine, Py_3_S_3_ = thiacalix[3].pyridine).^[^ ^13^, ^14^, ^15^ ^]^

	Mo—N bond length	N—Mo—N bond angle
[Mo(CO)_3_(TPn)] (4) [MoO_3_(TPn)] (5)	2.332 2.464	82.27 77.32
[Mo(CO)_3_(Py_3_S_3_)]^[^ ^15^ ^]^ [MoO_3_(Py_3_S_3_)]^[^ ^15^ ^]^	2.277 2.425	80.92 72.90
**[**Mo(CO)_3_(tolyl‐ACP)]^[^ ^13^, ^14^ ^]^ [MoO_3_(tolyl‐ACP)]^[^ ^13^, ^14^ ^]^	2.249 2.319	72.19 68.75

## 3 Conclusion

The dome‐shaped molybdenum(0) tricarbonyl complex [Mo(CO)_3_(TPn)] (**4**) supported by the new dithia‐[2.1.1]‐(2,6)‐pyridinophane ligand was synthesized, characterized, and deposited on an Au(111) surface. Monolayers of **4** were investigated by using various surface spectroscopic methods (IRRAS, XPS, and NEXAFS). While the intact complex could be measured under IRRAS conditions, partial loss of CO ligands due to X‐ray irradiation and concomitant oxidation of molybdenum were observed by XPS. The NEXAFS and IRRAS measurements indicated a slightly tilted orientation of the dome‐shaped complex in regard to the surface, especially based on the observation of the A’(1), A’’, and A’(2) vibrational modes of the carbonyl ligands in the IRRA spectrum. Moreover, an electronic influence of the gold substrate on the de/activation of the carbonyl ligands was evident from a shift to higher wavenumbers.

Notably, the ethylene bridge replacing a bridging sulfur atom of thiacalix[3](2,6)pyridine increases the size of the cavity and further stabilizes the Mo(0) metal center. Regarding an oxidation to a Mo(VI) oxo complex **5**, however, the macrocycle cannot retain all metal–nitrogen bonds to the oxidized metal center, cleaving one Mo‐pyridine bond. Whereas in a homogeneous solution this ultimately leads to demetallation, on the surface this conversion occurs to some extent, in contrast to the related thiacalix[3]pyridine system, which does not attain the Mo(VI) state. Regarding the propensity to form Mo(VI) oxo species on the surface, which is a prerequisite to use these systems as catalysts for oxygen transfer reactions,^[^ ^13^, ^14^ ^]^ the molybdenum(0) tricarbonyl complex **4** supported by the dithiapyridophane ligand **3** thus takes an intermediate position between the analogous aza‐ and thiacalix[3]pyridine systems.

## 4 Experimental Section

4.1

4.1.1

##### Materials and Methods

The commercially available starting materials were purchased from Sigma Aldrich, ABCR, TCI chemicals, or Fluorochem in reagent grade. Unless otherwise noted, these reagents were used without further purification. Oxygen‐ and/or moisture‐sensitive materials, especially the molybdenum(0) complexes, were handled using standard Schlenk techniques (nitrogen atmosphere dried over granulate P_4_O_10_) and a MBraun LABmaster glove box filled with argon (O_2_ < 1 ppm and H_2_O < 1 ppm). All solvents were of commercially available reagent grade. Dichloromethane, toluene, and *n*‐pentane were dried by heating to reflux under nitrogen or argon atmosphere over calcium hydride. Anhydrous deuterated solvents were freeze‐pump‐thaw degassed and dried over 4 Å molecular sieves. NMR spectra were recorded at 300 K with a Bruker AVANCE III HD pulse Fourier transform spectrometer equipped with a cryo‐probehead Prodigy BBO400S1 BB‐H&F‐D‐05‐Z operating at frequencies of 400.13 MHz (^1^H‐NMR) and 100.62 MHz (^13^C‐NMR). Referencing was performed using the solvent residue signal (5.32 ppm for CD_2_Cl_2_ and 7.28 ppm for CDCl_3_). Signals were assigned with the help of DEPT‐135 and 2D correlation spectra (^1^H, ^1^H‐COSY) correlation spectroscopy, (^1^H,^13^C‐HSQC) heteronuclear single quantum coherence, (^1^H,^13^C‐HMBC) heteronuclear multiple bond correlation. Infrared spectra were recorded at room temperature on a Bruker Alpha fourier transform infrared (FT‐IR) Spectrum with Platinum attenuated total reflection (ATR) setup or on a Bruker Vertex70 FT‐IR spectrometer using a broadband spectral range extension VERTEX FM for full mid and far IR in the range of 6.000–80 cm^−1^. Raman spectra were recorded at room temperature on a Bruker RAM II FT‐Raman spectrometer using a liquid nitrogen cooled, highly sensitive Ge detector, 1064 nm radiation, and 3 cm^−1^ resolution. Flash Column chromatography was carried out with a Biotage Isolera one spectra with UV detector (*λ* = 200–400 nm) and prepacked SNAP Ultra cartridges (different sizes). R_f_‐values were determined by thin‐layer chromatography on Polygram Sil G/UV254 (Macherey‐Nagel, 0.2 mm particle size) with a Comag UV lamp (*λ* = 254 nm). The elemental analyses were performed using an Elementar Vario MICRO cube element analyzer; the samples were prepared in tin vessels and were burnt in a stream of oxygen. High‐resolution electrospray ionisation mass spectra (HR‐ESI) were measured with a Thermo Scientific Q Exactive Plus.

##### Single Crystal Structure Determination

The data collections were performed with an XtaLAB Synergy, Dualflex, HyPix diffractometer using CuKα radiation (*λ* = 1.54184). The structures were solved with SHELXT^[^ ^56^ ^]^ and refined with SHELXL^[^ ^57^ ^]^ using Least Squares minimization. All nonhydrogen atoms were refined anisotropically. The C—H H atoms were positioned with idealized geometry and were refined isotropically with *U*_iso_(H) = 1.2 *U*_eq_(C) using a riding model. For compound **3** absolute structure was determined and is in agreement with the selected setting (Flack *x* = 0.012(19) by classical fit to all intensities and 0.024(24) from 1342 selected quotients (Parsons’ method). This crystal was pseudomerohedrally twinned. Both individuals were indexed separately, and finally, a twin refinement using data in HKLF‐5 format was performed. The asymmetric unit of 4 contains one additional dichloromethane solvate molecule.

CCDC‐ 2 431 887 (**3**) and CCDC‐ 2 431 888 (**4**) contain the supplementary crystallographic data for this article. These data can be obtained free of charge from the Cambridge Crystallographic Data Centre via http://www.ccdc.cam.ac.UK/data_request/cif.

##### Preparation of Surface

For the IRRAS measurements, glass substrates with a 50 Å titanium adlayer and a 200 nm evaporated gold film from EMF corporation (Ithaca, NY) were used. XPS and NEXAFS measurements were undertaken on sputtered Au/Cr/Si wafers (Au 200 nm, Cr 10 nm). Before each preparation, the gold wafer was cleaned by flame annealing with butane gas. Monolayers were prepared by immersing Au(111) substrates in 0.5 mM solutions of the respective compound in dry dichloromethane at room temperature under nitrogen or argon atmosphere. After 1 h of immersion, the sample was removed from the solution, rinsed with dry dichloromethane, and dried in a stream of nitrogen or argon gas.

##### IRRAS

The surface adsorbed molecules were investigated by using a Bruker VERTEX 70 FT‐IR spectrometer equipped with a polarization modulation accessory (PMA) 50 unit (Bruker Optik GmbH, Ettlingen, Germany). This instrument allows recording IRRAS and PM‐IRRAS data with a spectral range from 4000 down to 750 cm^−1^. IRRAS data were collected with a liquid nitrogen‐cooled mercury cadmium telluride (MCT) detector in a horizontal reflection unit for grazing incidence (Bruker A518). The sample chamber was purged with dry nitrogen before and during measurements. An undeuterated hexadecane‐thiol SAM on Au(111) was used as a reference for the background spectrum for conventional IRRA spectra. Each spectrum contains 2048 averaged spectra. A *p*‐polarized beam at an incident angle of 80° to the surface normal was used for measurements. All spectra were recorded with 4 cm^−1^ resolution. PM‐IRRAS data were collected with the PMA 50 accessory using a liquid nitrogen‐cooled MCT detector. The photoelastic modulator (PEM) maximum efficiency was set for the half‐wave at 1750 cm^−1^ for analysis of the area from 2000 to 1000 cm^−1^. All spectra were recorded with 4 cm^−1^ resolution. Air‐sensitive samples were measured with a specially designed inert‐gas cell.^[^ ^58^ ^]^ In addition to an exposure window, this cell also has two hose connections to change the atmosphere. Processing of IRRAS and PM‐IRRAS data was carried out using the OPUS software Version 6.5 (Bruker, Germany). Baseline correction of the resulting IRRAS data was performed by the rubber band method in an interactive mode. PM‐IRRAS data were processed by the implicit removal of the Bessel function through manual baseline correction.

##### XPS and NEXAFS

The XPS and NEXAFS measurements were performed at the BESSY II synchrotron radiation facility using the PREVAC end station at the beamline HE‐SGM. The experimental station is equipped with a hemispherical VG Scienta R3000 photoelectron analyzer. The energy resolution E/DE of the beamline with 150 mm slits is 800. XP survey spectra were secured at 700 eV photon energy using an analyzer pass energy of 100 eV, whereas for the C 1s and Mo 3d spectra, the photon energy used was 350 eV with a pass energy of 50 eV. For N 1s spectra, the photon energy was at 500 eV with a pass energy of 50 eV, and for O 1s the photon energy was at 650 eV with a pass energy of 50 eV. All spectra were acquired at normal electron emission. For the determination of the relative composition, the XP spectra were energy‐corrected using the Au 4f_7/2_ line at a binding energy of 84.0 eV as reference. Background correction was performed using a combination of a Shirley and a linear background for all signals. Peak fitting was performed using the program CasaXPS. The fitting parameters are shown in the Supporting Information. The samples were transported via a transfer box from the nitrogen glovebox to the beamline to protect the samples from contact with oxygen. To correct the photon flux of the NEXAFS measurements, all spectra were divided by the spectrum obtained for a freshly sputtered clean gold substrate and then edge‐step normalized (using the average intensities for the C K‐edge between 275 ± 0.5 eV and 320 ± 0.5 eV, for the N K‐edge between 395 ± 0.5 eV and 420 ± 0.5 eV, and for the O K‐edge between 525 ± 0.5 eV and 560 ± 0.5 eV as pre‐ and post‐edge).

##### Powder Diffraction

Powder X‐ray diffraction (XRPD) was performed in transmission geometry using a PANalytical Empyrean (Cu K_*α*_ radiation, focusing X‐ray mirror, PIXcel 1‐D detector).

##### Computational Details

All DFT calculations were carried out using the ORCA 4.2.1 program package.^[^ ^59^ ^]^ The geometry optimizations and the calculation of vibrational modes were performed by using the B3LYP functional^[^ ^60^ ^]^ and the Ahlrichs def2‐TZVP basis set^[^ ^61^, ^62^ ^]^ in conjunction with the chain of spheres approximation (RIJCOSX), the general Ahlrichs Coulomb fitting basis set denoted def2/J.^[^ ^63^, ^64^ ^]^ In addition to account for the dispersion effects, Grimme's dispersion correction with Becke–Johnson damping (D3BJ)^[^ ^65^ ^]^ was used. The valence electronic spectra were calculated by using time‐dependent density functional theory (TDDFT) with B3LYP,^[^ ^60^ ^]^ the def2‐TZVPP basis set,^[^ ^61^ ^]^ the RIJCOSX approximation^[^ ^63^ ^]^ and fine numerical integration grids (grid6 and gridX6 in ORCA nomenclature). The restriction of the orbital window from which electrons were to be excited to the 1s orbital of the atom of interest resulted in an enhancement of the integral accuracy. A total of 80 roots with electric quadrupole contributions were computed. This process was repeated for each unique carbon and nitrogen atom in the molecule.

##### Synthesis

2,6‐dimercaptopyridine (**1**)^[^ ^26^, ^27^ ^]^ and 1,2‐bis(6‐chloropyridin‐2‐yl)ethane (**2**)^[^ ^22^ ^]^ were synthesized according to literature procedures.

##### Synthesis: Dithia‐2.1.1‐(2,6)‐Pyridinophane (3)

2,6‐dimercaptopyridine (286 mg, 2 mmol) and 1,2‐Bis(6‐chloropyridin‐2‐yl)ethane (506 mg, 2 mmol) were dissolved in 50 mL toluene and heated to reflux at 110 °C for 5d. Afterwards, 20 mL of deionized water water was added to the mixture. The organic phase was separated, and the aqueous phase was extracted with dichloromethane (3 × 30 mL). The combined org. phases were dried over magnesium sulfate, and the solvent was removed in vacuo. The crude product was purified by column chromatography on silica gel (thin layer chromatography, (TLC): dichloromethane/methanol (1:1), *R*_f_ = 0.85) as well as recrystallization (acetone) to obtain the desired product as colorless crystals (257 mg, 794 μmol, 40%). ^**1**^ **H NMR** (400.13 MHz, CDCl_3_): *δ* = 7.49 (dd, ^3^ *J* = 7.5 Hz, ^3^ *J* = 8.0 Hz, 1H, *H*‐3), 7.34 (t, ^3^ *J* = 7.7 Hz, 2H, *H*‐8, *H*‐15), 7.21 (d, ^3^ *J* = 7.7 Hz, 2H, *H*‐2, *H*‐4), 7.10 (dd, ^4^ *J* = 1.0 Hz ^3^ *J* = 7.7 Hz, 2H, *H*‐7, *H*‐16), 6.82 (dd, ^4^ *J* = 1.0 Hz ^3^ *J* = 7.7 Hz, 2 H, *H*‐9, *H*‐14), 3.24 (s, 4H, *H*‐11, *H*‐12) ppm; ^**13**^ **C NMR** (100.62 MHz, CDCl_3_): *δ* = 160.1 (*C*‐10, *C*‐13), 157.3 (*C*‐1, *C*‐5), 154.1 (*C*‐6, *C*‐17), 137.0 (*C*‐3), 136.8 (*C*‐8, *C*‐15), 124.4 (*C*‐9, *C*‐14), 123.2 (*C*‐2, *C*‐4), 121.7 (*C*‐7, *C*‐16), 37.1 (*C*‐11, *C*‐12) ppm; **IR** (neat): 3042 (br, w), 2916 (br, w), 2846 (br, vw), 1576 (m), 1556 (s), 1544 (s), 1431 (m), 1409 (s), 1374 (m), 1239 (w), 1156 (m), 1119 (s), 1078 (m), 980 (m), 835 (m), 776 (s), 725 (m), 674 (m), 653 (m), 610 (w), 588 (w), 549 (w), 524 (vw), 494 (w), 463 (w), 425 (w) cm^−1^; **FT‐Raman** (solid): 3053 (s), 3023 (m), 2953 (m), 2919 (s), 1581 (m), 1560 (s), 1545 (s), 1443 (m), 1413 (m), 1379 (m), 1344 (w), 1321 (w), 1242 (s), 1196 (m), 1178 (s), 1140 (s), 1076 (s), 993 (s), 982 (vs), 937 (w), 837 (m), 793 (m), 744 (w), 731 (w), 679 (s), 658 (s), 550 (w), 526 (s), 498 (w), 469 (w), 442 (w) cm^−1^, **HRMS (ESI)**: *m/z* calcd for C_17_H_13_N_3_S_2_+H^+^: 324.0624 [*M*+H]^+^; found: 324.0613; **elemental analysis** calcd (%) for C_17_H_13_N_3_S_2_: C 63.13, H 4.05, N 12.99; S 19.82, found: C 62.94, H 3.96, N 13.08, S 19.84.

##### Synthesis: [Mo(CO)3(TPn)] (4)

Under inert atmosphere, dithia‐2.1.1‐(2,6)‐pyridinophane (**3**) (60 mg, 186 μmol) and the precursor [Mo(CO)_3_(cht)] (50.5 mg, 186 μmol) were dissolved in 6 mL dry dichloromethane. The dark red solution was stirred overnight at room temperature. The suspension was filtered, and the precipitate was washed with 2 mL dry dichloromethane, 2 mL dry toluene, and 10 mL dry *n*‐pentane. The product was dried under vacuum and was obtained as a red powder (60 mg, 119 μmol, 64%). Crystals suitable for single‐crystal X‐ray diffraction were obtained by slow evaporation of the product in dichloromethane under argon atmosphere at room temperature. ^**1**^ **H NMR** (400.13 MHz, CD_2_Cl_2_): *δ* = 7.58 (dd, ^3^ *J* = 6.6 Hz, ^3^ *J* = 8,7 Hz, 1H, *H*‐3), 7.52–7.41 (m, 6H, *H*‐2, *H*‐4, *H*‐8, *H*‐9, *H*‐14, *H*‐15), 7.21 (dd, ^4^ *J* = 1.6 Hz, ^3^ *J* = 7.5 Hz, 2H, *H*‐7, *H*‐16), 4.42–4.35 (m, 2H, *H*‐11, *H*‐12), 3.42‐3.35 (m, 2H, *H*‐11, *H*‐12) ppm; ^**13**^ **C NMR** (100.62 MHz, CD_2_Cl_2_): *δ* = 165.0 (*C*‐10, *C*‐13), 160.8 (*C*‐1, *C*‐5), 151.9 (*C*‐6, *C*‐17), 138.3 (*C*‐2, *C*‐4 or *C*‐7, *C*‐16 or *C*‐8, *C*‐15), 137.3 (*C*‐3), 128.8 (*C*‐2, *C*‐4 or *C*‐7, *C*‐16 or *C*‐8, *C*‐15), 125.3 (*C*‐2, *C*‐4 or *C*‐7, *C*‐16 or *C*‐8, *C*‐15), 126.9 (*C*‐9, *C*‐14), 37.1 (*C*‐11, *C*‐12) ppm; **IR** (KBr): 3063 (br, vw), 2935 (br, vw), 1895 (vs), 1767 (vs),1753 (vs), 1581 (m), 1569 (m), 1554 (m), 1537 (m), 1446 (m), 1435 (m), 1418 (m), 1403 (m), 1381 (m), 1309 (m), 1262 (m), 1243 (m), 1224 (m), 1177 (s), 1150 (m), 1123 (m), 1019 (m), 999 (m), 985 (m), 969 (m), 918 (m), 908 (m), 898 (m), 809 (s), 751 (m), 735 (s), 706 (m), 662 (m), 635 (s), 623 (m), 609 (m), 590 (m), 543 (m), 531 (m), 487 (s), 463 (m), 440 (m), 427 (m) cm^−1^; **FT‐Raman** (solid): 3118 (vw), 3066 (w), 3053 (vw), 3034 (vw), 2985 (vw), 2937 (w), 1887 (s), 1776 (m), 1768 (m), 1581 (m), 1570 (m), 1554 (w), 1421 (w), 1402 (w), 1383 (w), 1365 (w), 1309 (vw), 1263 (w), 1227 (vw),1169 (w), 1149 (w), 1126 (vw), 1103 (vw), 1018 (w), 1001 (s), 966 (w), 833 (vw), 810 (vw), 735 (w), 705 (w), 685 (m), 673 (m), 636 (w), 596 (vw), 507 (s), 486 (m), 453 (w), 443 (w), 428 (w) cm^−1^; **HRMS (ESI)**: *m/z* calcd for C_20_H_13_N_3_S_2_O_3_Mo: 504.94469 [*M*]^+^; found: 504.94383, **elemental analysis** calcd (%) for C_20_H_13_N_3_S_2_O_3_Mo·1·CH_2_Cl_2_: C 42.87, H 2.57, N 7.14; S 10.90, found: C 42.61, H 2.85, N 7.48, S 11.04.

## Conflict of Interest

The authors declare no conflict of interest.

## Supporting information

Supplementary Material

## Data Availability

The data that support the findings of this study are available in the supplementary material of this article.
