# Supplementary material for: Surface Deposition of Dome‐Shaped Metal‐Organic Complexes: A New Approach to the Generation of Single‐Site Catalysts
Source: Chempluschem. 2025 Jun 28;90(8):e202500274. doi: 10.1002/cplu.202500274 (PMC12352724; doi:10.1002/cplu.202500274)
Supplement: Supplementary file 1 — Supplementary Material [file CPLU-90-e202500274-s001.pdf]

Supporting Information

Surface Deposition of Dome-Shaped Metal-Organic Complexes: A New Approach to the Generation of Single-Site Catalysts

Küpra Yildiz,[a] Kai Uwe Clausen,[a] Christian Näther,[a] Thomas Strunskus,[b] and Felix Tuczek*[a]

[a] Institute of Inorganic Chemistry, Christian-Albrechts-University of Kiel, Germany

[b] Department of Material Science, Christian-Albrechts-University of Kiel, Germany

Table of Contents

1. Experimental Section 4
1.1. NMR spectroscopy of dithia-2.1.1-(2,6)-pyridinophane (3) 4
1.2. Mass spectrometry of dithia-2.1.1-(2,6)-pyridinophane (3) 5
1.3. IR and Raman spectroscopy of dithia-2.1.1-(2,6)-pyridinophane (3) 5
1.4. NMR spectroscopy of [Mo(CO)$_3$(TPn)] (4) 6
1.5. Mass spectrometry of [Mo(CO)$_3$(TPn)] (4) 7
1.6. XRD pattern of [Mo(CO)$_3$(TPn)] (4) 7
2. Single Crystal X-Ray Structure Determination 8
3. Analysis of Molecule Structure of [Mo(CO)$_3$(TPn)] (4) 10
4. Infrared Reflection Absorption Spectroscopy (IRRAS) 11
4.1. PM- IRRA spectroscopy of [Mo(CO)$_3$(TPn)] (4) 11
4.2. PM-IRRA spectroscopy of [Mo(CO)$_3$(TPn)] (4) with Bessel function 11
4.3. Orientation of the transition dipole moments of the CO vibrational modes 12
4.4. Vibrational analysis of [Mo(CO)$_3$(TPn)] (4) 12
5. X-Ray Photoelectron Spectroscopy (XPS) 15
5.1. XPS measurements of [Mo(CO)$_3$(TPn)] (4) on Au(111) 15
5.1.1. Monolayer on Au(111) 15
5.1.2. Thicklayer on Au(111) 16
5.2. XPS measurements of dithia-2.1.1-(2,6)-pyridinophane (3) on Au(111) 17
5.2.1. Monolayer on Au(111) 17
5.2.2. Thicklayer on Au(111) 18
6. Reactivity toward oxygen (O_2) 20
7. Computational Details 22
7.1. Coordinates of DFT-calculated structures of [Mo(CO)$_3$(TPn)] (4) 22
7.2. Coordinates of DFT-calculated structures of [MoO$_3$(TPn)] (5) 23
7.3. Calculation of IRRA spectrum of [Mo(CO)$_3$(TPn)] (4) 24

8. Literature 25

1. Experimental Section

1.1. NMR spectroscopy of dithia-2.1.1-(2,6)-pyridinophane (3)

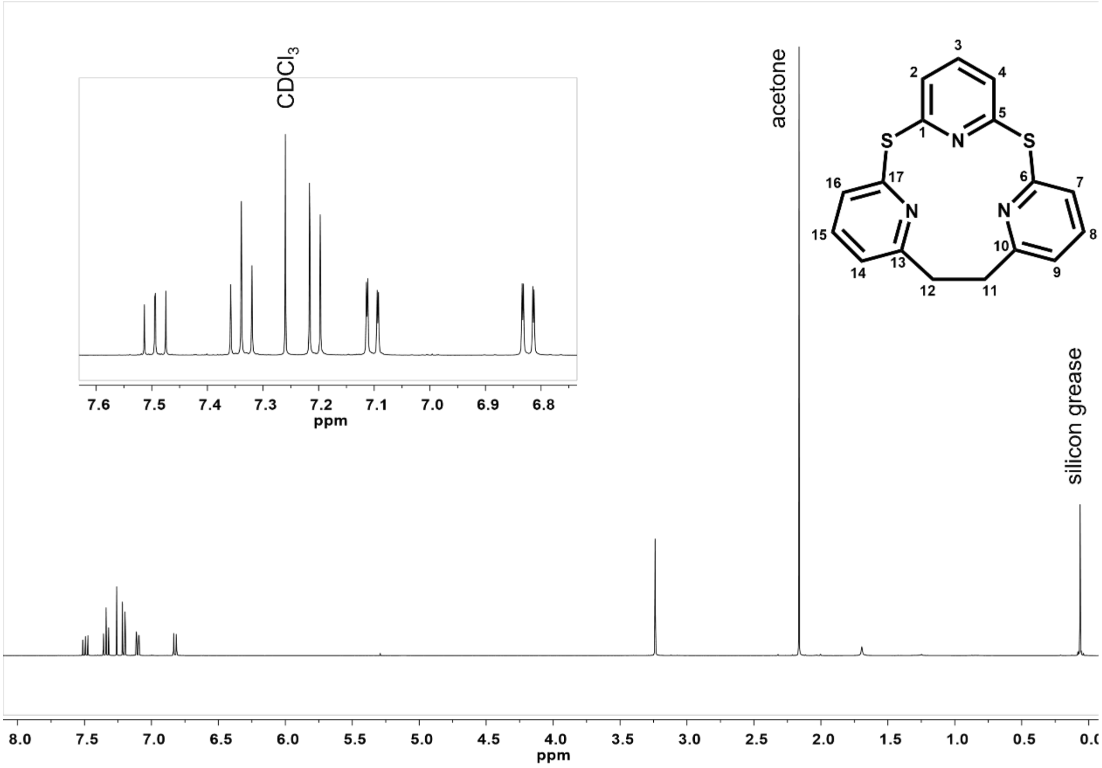

Figure S1: ^{1}H NMR spectrum of dithia-2.1.1-(2,6)-pyridinophane (**3**) measured in $CDCl_3$ at 300 K.

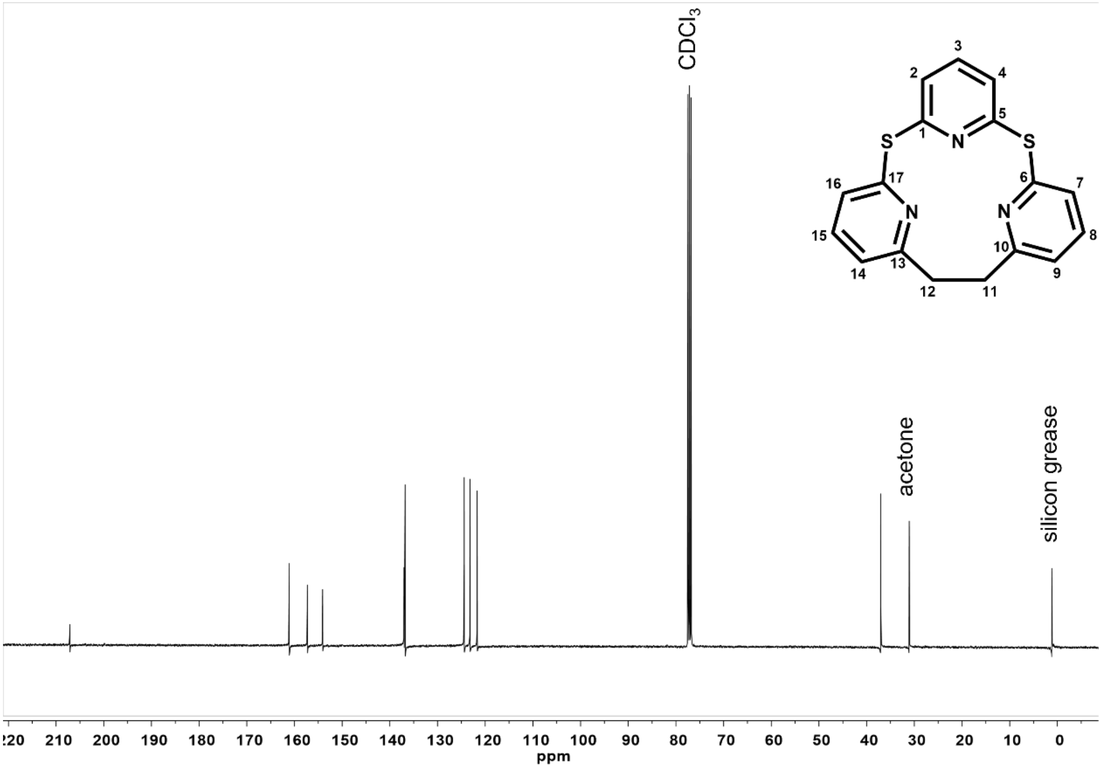

Figure S2: ^{13}C NMR spectrum of dithia-2.1.1-(2,6)-pyridinophane (**3**) measured in $CDCl_3$ at 300 K.

1.2. Mass spectrometry of dithia-2.1.1-(2,6)-pyridinophane (3)

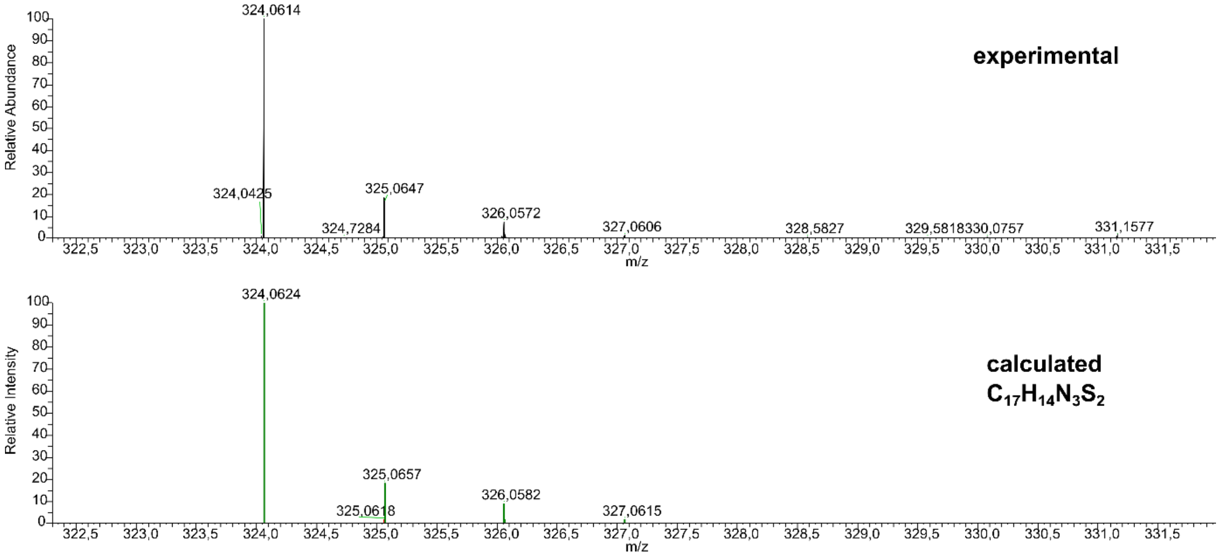

Figure S3: Mass spectrum of dithia-2.1.1-(2,6)-pyridinophane (TPn, **3**) with the isotopic pattern of the molecular peak $[M\text{-}H]^+$.

1.3. IR and Raman spectroscopy of dithia-2.1.1-(2,6)-pyridinophane (3)

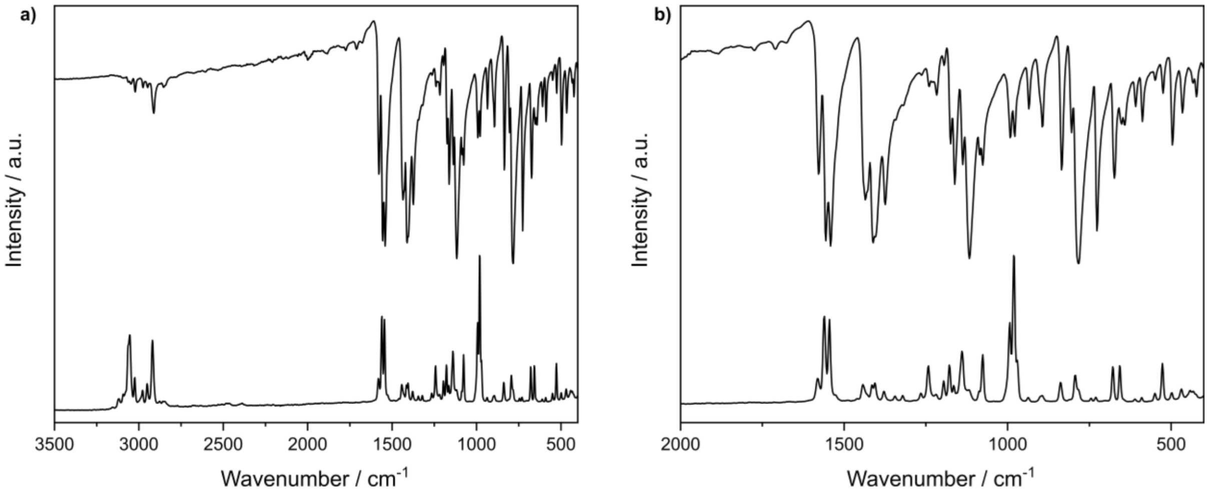

Figure S4: a) IR and Raman spectra of the dithia-2.1.1-(2,6)-pyridinophane (**3**) between 3500 cm^{-1} and 400 cm^{-1}. The Raman data were multiplied by factor of 2; **b)** Enlargement of the vibrational region between 2000 cm^{-1} and 400 cm^{-1}.

1.4. NMR spectroscopy of [Mo(CO)3(TPn)] (4)

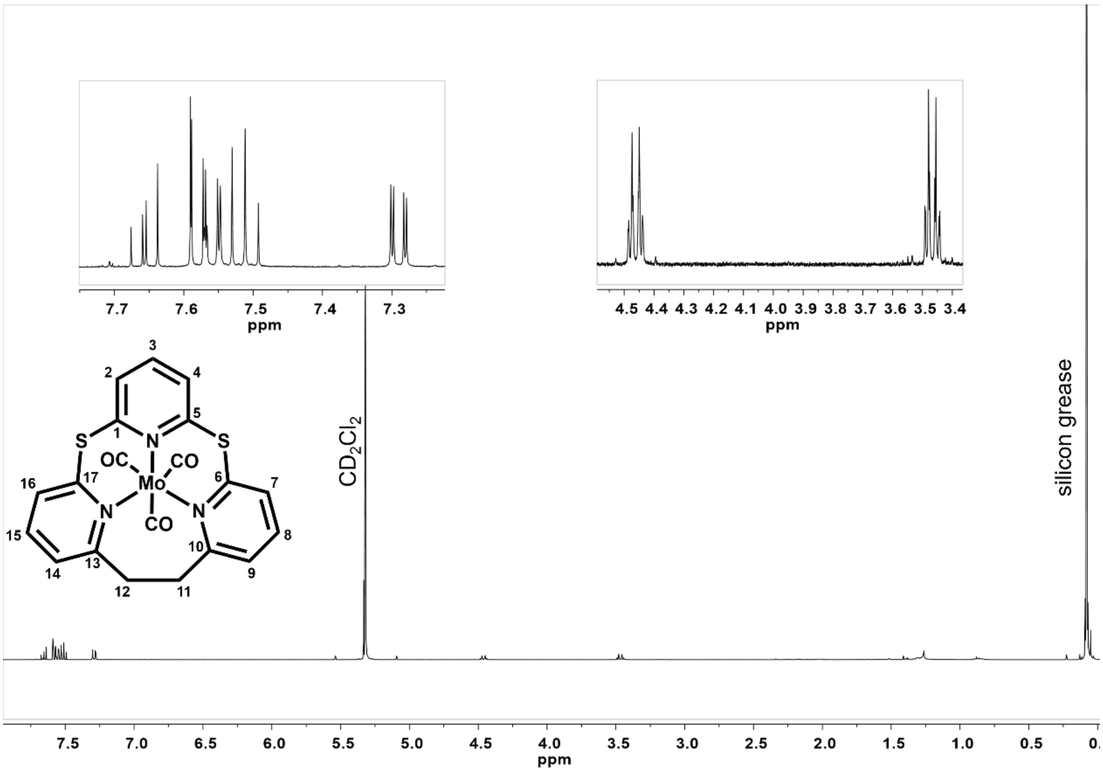

Figure S5: ^{1}H NMR spectrum of [Mo(CO)$_3$(TPn)] (**4**) measured in CD_2Cl_2 at 300 K.

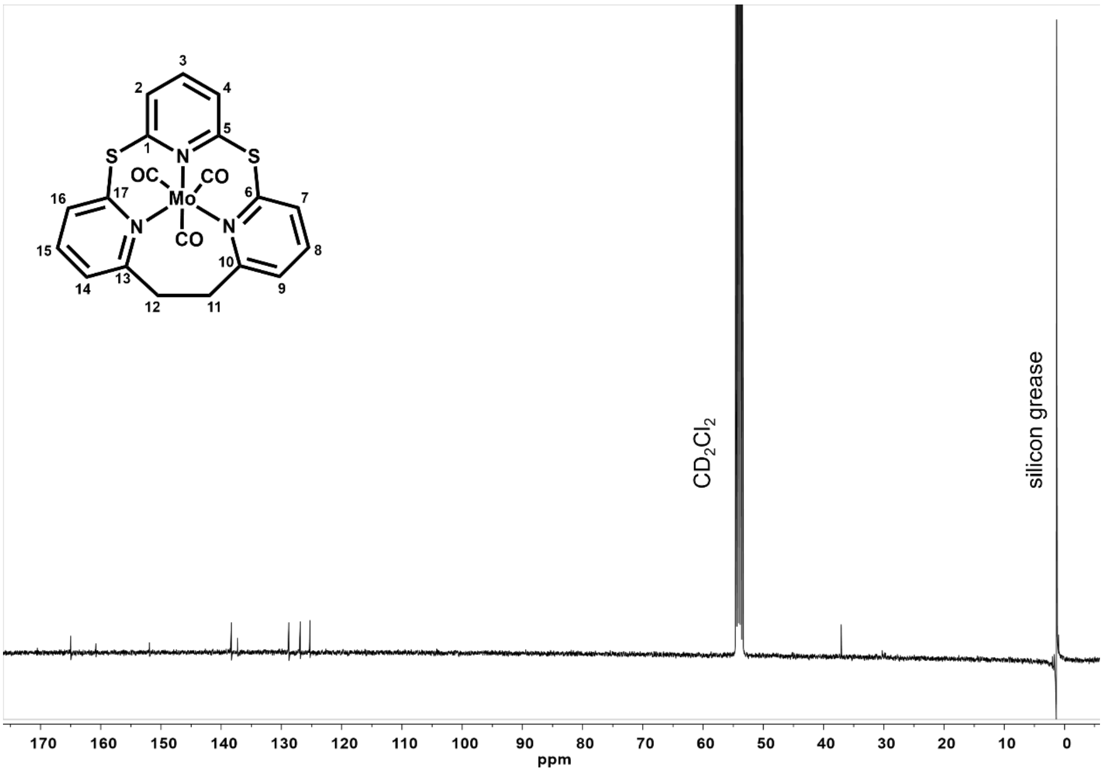

Figure S6: ^{13}C NMR spectrum of [Mo(CO)$_3$(TPn)] (**4**) measured in CD_2Cl_2 at 300 K.

1.5. Mass spectrometry of [Mo(CO)3(TPn)] (4)

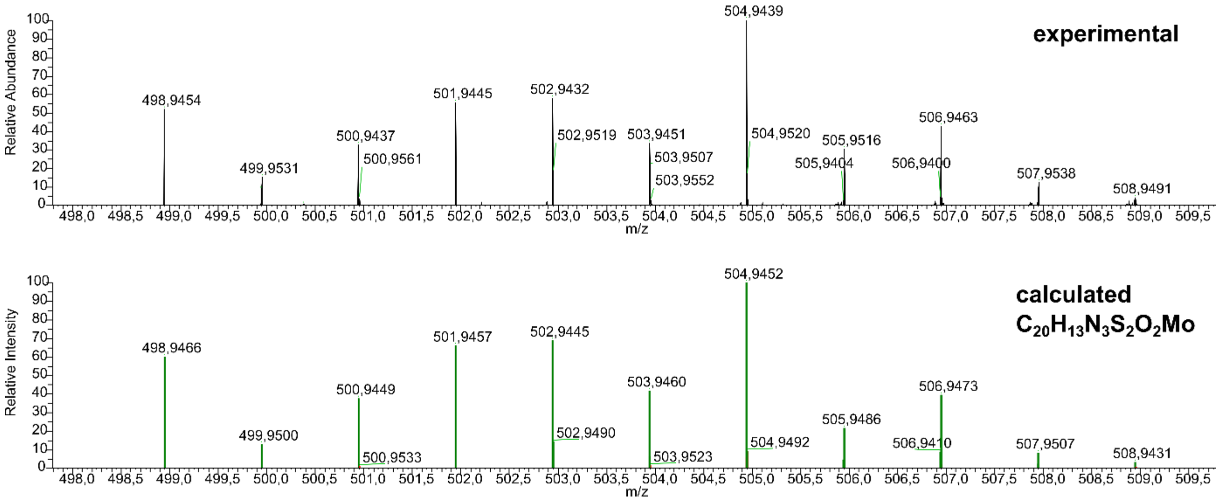

Figure S7: Mass spectrum of [Mo(CO)3(TPn)] (**4**) with the isotopic pattern of the molecular peak [M]+.

1.6. XRD pattern of [Mo(CO)3(TPn)] (4)

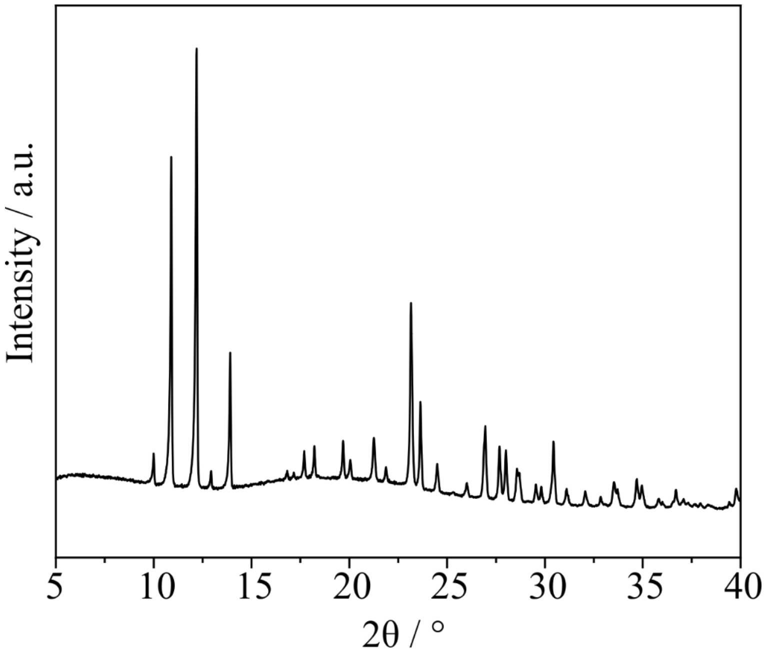

Figure S8: XRD pattern spectrum from 5° to 40° from the red powder of [Mo(CO)3(TPn)] (**4**).

2. Single Crystal X-Ray Structure Determination

Table S1: Crystal data and structure refinement for ligand **3** and complex **4**.

Compound	**3**	**4**
Empirical formula	$C_{17}H_{13}N_3S_2$	$C_{21}H_{15}Cl_2MoN_3O_3S_2$
Formula weight	323.42	588.32
Temperature/K	100(2) K	100.0(1)
Crystal system	monoclinic	monoclinic
Space group	*Cc*	$P2_1m$
a/Å	14.9953(3)	9.4056(2)
b/Å	8.65433(18)	12.6728(2)
c/Å	11.5366(3)	9.6753(2)
α/°	90	90
β/°	90.9083(19)	113.855(2)
γ/°	90	90
Volume/Å^3	1496.96(6)	1054.73(4)
Z	4	2
ρ_{calc}g/cm^3	1.435	1.852
μ/mm^{-1}	3.205	9,547
Radiation	Cu-Kα	Cu-Kα
Crystal size/mm	0.25 × 0.20 × 0.03	0.15 × 0.10 × 0.06
2Θ-max/°	159.15	159.75
Reflections collected	3362	13614
R_{int}	-	0.0260
Independent reflections	3362	2367
Reflections with [I>=2σ (I)]	3355	2346
Parameters	200	158
Goodness-of-fit on F^2	1.051	1.094
R1 for all [I>=2σ (I)]	0.0335	0.0348
WR2 for all refl.	0.0974	0.0919
Largest diff. peak/hole / e Å^{-3}	0.29/-0.25	1.59/-1.44

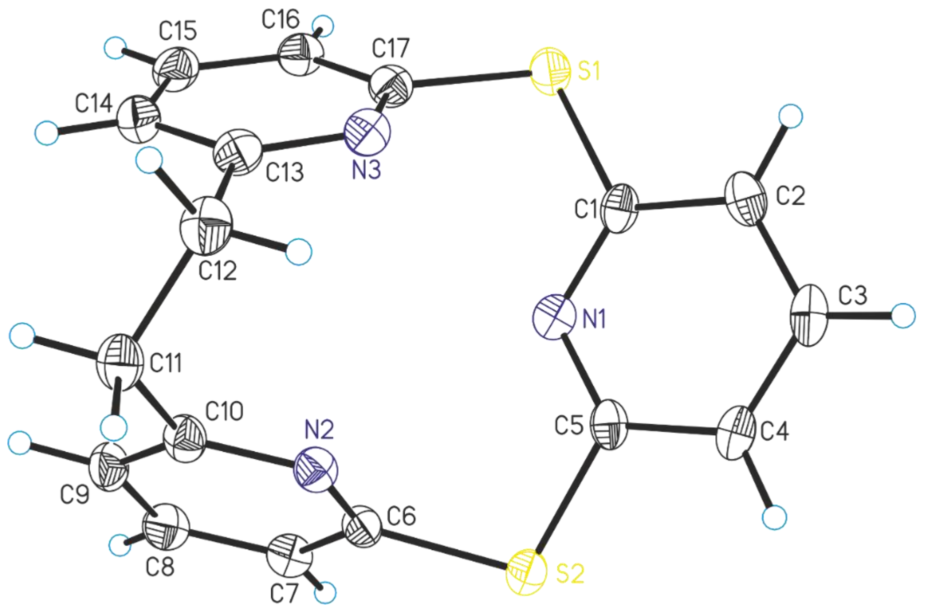

Figure S9: Crystal structure of ligand **3** with labeling and displacement ellipsoids drawn at the 50% probability level.

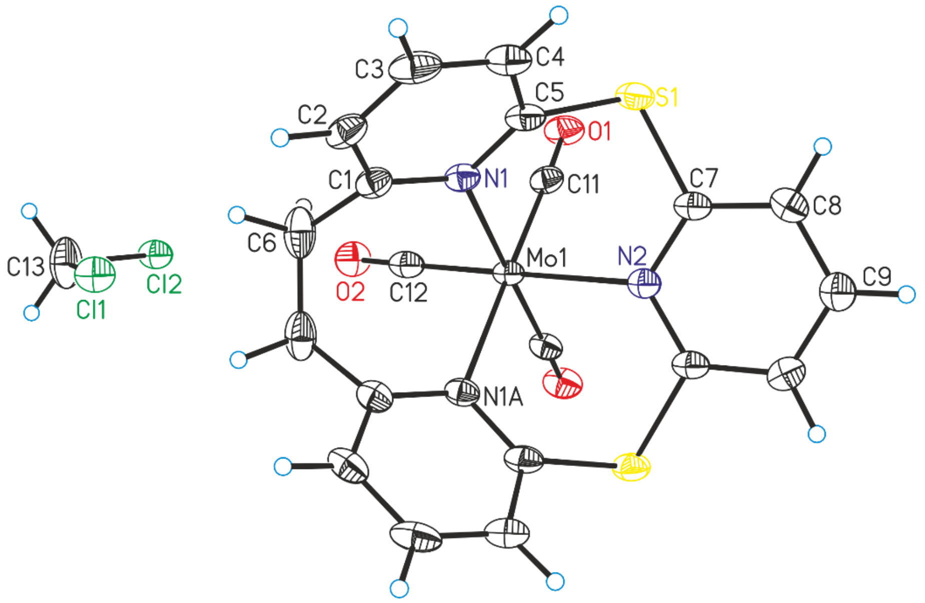

Figure S10: Crystal structure of complex **4** with labeling and displacement ellipsoids drawn at the 50% probability level. Please not that the complex is located on a crystallographic mirror plane and that the formula unit contain one dichloromethane solvate molecule.

3. Analysis of Molecule Structure of [Mo(CO)$_3$(TPn)] (4)

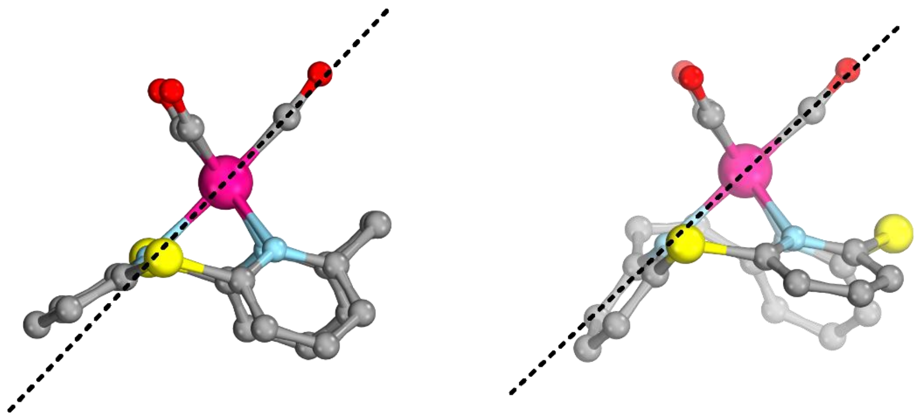

Figure S11: Detailed view of the molecule structure of [Mo(CO)$_3$(TPn)] (**4**) with an axis drawn from the carbonyl C *via* the molybdenum to the *trans*-N to demonstrate the upwards or downwards deviation of pyridines, respectively.

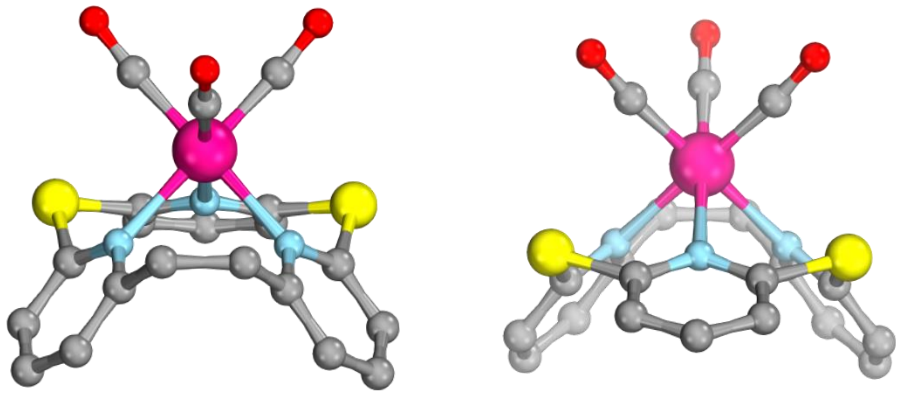

Figure S12: Detailed view of the molecule structure of [Mo(CO)$_3$(TPn)] (**4**) along the ethylene-bridge and along the *trans*-N of the pyridine opposite to the ethylene-bridge to demonstrate the position of pyridines, respectively.

4. Infrared Reflection Absorption Spectroscopy (IRRAS)

4.1.PM- IRRA spectroscopy of [Mo(CO)$_3$(TPn)] (4)

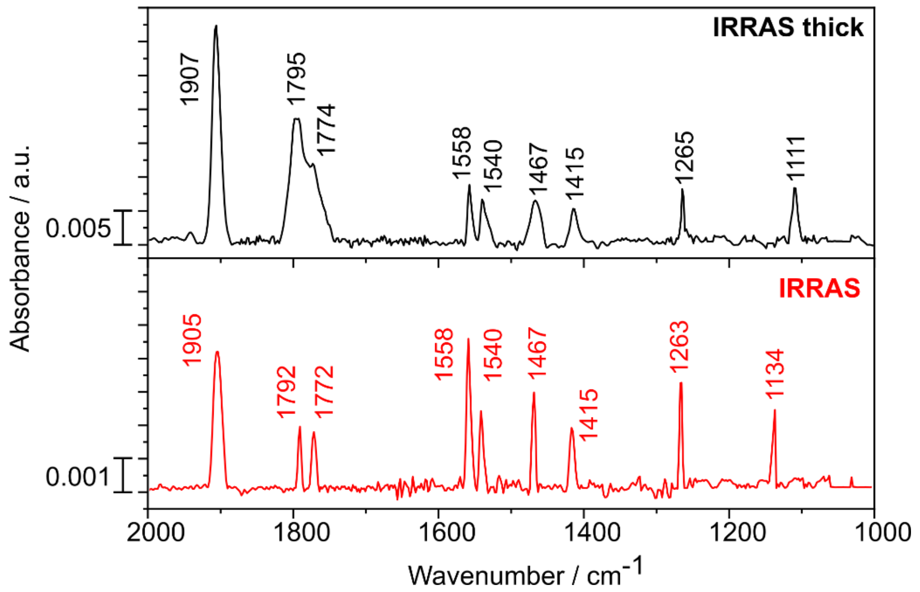

Figure S13: a) Measured PM-IRRA spectra of **4** as a thicker layer and as a monolayer adsorbed on a Au(111) surface.

4.2. PM-IRRA spectroscopy of [Mo(CO)$_3$(TPn)] (4) with Bessel function

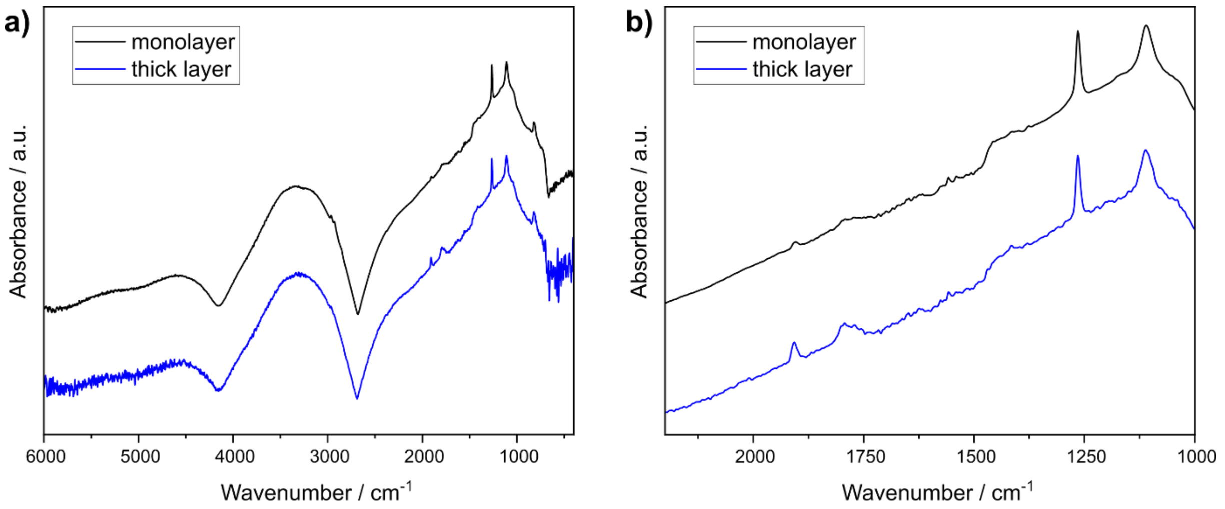

Figure S14: a) Measured PM-IRRA spectra of **4** as a thicker layer and as a monolayer adsorbed on a Au(111) surface with Bessel function between 6000 cm^{-1} and 400 cm^{-1}. **b)** Enlargement of the spectra between 2200 cm^{-1} and 1000 cm^{-1}.

4.3. Orientation of the transition dipole moments of the CO vibrational modes

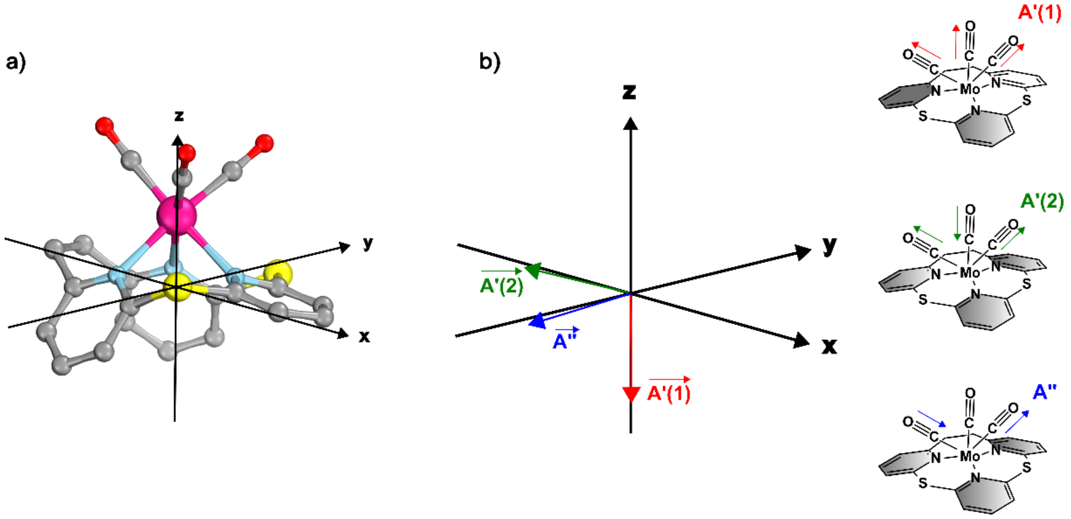

Figure S15: a) Illustrated position of [Mo(CO)$_3$(TPn)] (**4**) in a coordinate system, assuming that the plane through the three nitrogen donor atoms is oriented in the xy plane. **b)** Orientation of transition dipole moment (TDM) vectors of the three carbonyl vibration modes (A'(1), A'(2) and A''). The TDM vectors were calculated by DFT for the IRRA spectroscopy.

4.4. Vibrational analysis of [Mo(CO)$_3$(TPn)] (4)

Table S2: A full vibrational analysis with the assignment respectively is shown from 3000 cm^{-1} to 800 cm^{-1} for the carbonyl complex **4**. The energies of the calculated spectrum were corrected by using the equation: $V_{corr.} = (V_{calc.} / 1.22154) + 238.64$ cm^{-1}.

Mode	Calc. freq. uncorr.	Calc. freq. corr.	Calc. Int. IR.	Calc. Int. IRRAS	Bulk IR	IRRAS	TDM	Assignment
1	682.2	797.1	3.17	3.23			/	In plane deformation
2	693.2	806.1	0.84	0.00			=	In plane deformation
3	696.2	808.6	6.83	6.72			⊥	In plane deformation
4	722.5	830.1	6.47	0.00			=	In plane deformation
5	747.8	850.8	19.37	17.51	832		⊥	asym. C-H wagging
6	767.4	866.9	7.76	3.18			/	sym. C-H wagging
7	772.8	871.3	47.05	0.00			=	asym. C-H wagging
8	788.0	883.7	8.08	0.01	898		=	asym. C-H wagging
9	798.2	892.1	17.93	14.98			⊥	asym. C-H wagging
10	817.4	907.8	3.20	0.00			=	In plane deformation
11	827.7	916.2	11.56	11.52	907		⊥	sym. C-H wagging
12	833.4	920.9	5.70	0.00			=	In plane deformation
13	857.5	940.6	7.82	0.67			/	In plane deformation
14	909.5	983.2	0.32	0.00			=	asym. C-H wagging
15	912.7	985.8	0.44	0.00			=	asym. C-H wagging
16	932.9	1002.4	0.12	0.00			=	asym. C-H wagging
17	949.8	1016.1	1.07	0.00			=	asym. C-H wagging
18	980.5	1041.3	9.49	0.00			=	asym. C-H wagging ethyl-bridge
19	1003.5	1060.1	0.11	0.04			=	asym. C-H wagging
20	1011.1	1066.4	1.36	1.44			/	asym. C-H wagging
21	1011.7	1066.8	0.29	0.00			=	asym. C-H wagging
22	1014.5	1069.1	8.37	4.79			⊥	In plane deformation
23	1020.7	1074.2	4.32	0.00			=	In plane deformation

4.4. Vibrational analysis of [Mo(CO)3(TPn)] (4)

24	1020.9	1074.4	11.39	11.67	1105		⊥	In plane deformation
25	1031.5	1083.1	4.04	0.00			=	C-C stretching ethyl-bridge
26	1105.7	1143.8	2.74	0.04			=	C-H bending
27	1116.8	1152.9	2.19	2.04			/	C-H bending
28	1119.8	1155.4	2.42	0.00			=	C-H bending
29	1148.8	1179.1	13.78	6.59	1177		⊥	C-H bending
30	1150.9	1180.8	4.36	0.00			=	C-H bending
31	1159.4	1187.8	0.51	0.00			=	C-H bending
32	1174.5	1200.1	40.80	3.67	1203	1201	⊥	In plane deformation
33	1180.8	1205.3	14.84	0.00			=	C-H bending
34	1182.6	1206.7	4.66	2.45			⊥	C-H bending
35	1187.9	1211.1	12.42	0.00	1225		=	In plane deformation
36	1216.4	1234.4	12.33	4.36	1233		⊥	C-H bending ethyl-bridge
37	1241.3	1254.8	1.65	0.00			=	C-H bending ethyl-bridge
38	1262.4	1272.1	6.90	0.00			=	C-H bending ethyl-bridge
39	1284.2	1289.9	7.23	7.25	1262	1264	⊥	C-H bending ethyl-bridge
40	1299.7	1302.7	3.17	0.96			/	C=N=C stretching
41	1308.1	1309.5	0.001	0.00			=	C=N=C stretching
42	1313.8	1314.2	0.0003	0.00			=	C=N=C stretching
43	1332.6	1329.5	4.82	2.02			/	asym. C=C stretching ethyl-bridge
44	1384.6	1372.1	5.85	0.00	1380		=	sym. C=C stretching ethyl-bridge
45	1412.4	1394.9	58.75	0.82	1402		/	C=N=C stretching
46	1420.8	1401.7	36.86	0.00			=	C=N=C stretching
47	1429.4	1408.8	63.76	56.88	1417	1414	⊥	C=N=C stretching
48	1443.1	1420.0	25.42	0.00	1433		=	asym. C=C stretching
49	1450.8	1426.3	34.49	0.00			=	asym. C=C stretching
50	1459.8	1433.7	0.64	0.18			/	asym. C=C stretching
51	1472.6	1444.2	10.55	0.00	1446		=	sym. C=C stretching
52	1479.2	1449.5	79.91	2.18			/	asym. C=C stretching
53	1590.2	1540.4	17.62	0.00	1536		=	asym. C=C stretching
54	1599.9	1548.5	22.27	3.83	1552	1539	⊥	sym. C=C stretching
55	1603.3	1551.2	6.41	0.00			=	asym. C=C stretching
56	1614.7	1560.5	3.39	1.42	1568	1558	/	asym. C=C stretching
57	1629.3	1572.4	1.14	0.00	1580		=	asym. C=C stretching
58	1634.0	1576.3	16.69	0.63			/	asym. C=C stretching
59	1938.5	1825.5	1051.93	69.46	1753	1772	/	CO stretching A'(2)
60	1975.8	1856.1	892.50	0.00	1767	1792	=	CO stretching A''
61	2035.1	1904.7	1906.02	1884.96	1895	1905	⊥	CO stretching A'(1)
62	3063.1	2746.2	14.22	0.00	2933		=	asym. C-H stretching ethyl-bridge
63	3072.7	2754.1	51.30	1.76			/	sym. C-H stretching ethyl-bridge
64	3126.3	2798.0	0.72	0.00			=	asym. C-H stretching ethyl-bridge
65	3134.2	2804.4	7.68	0.26			/	sym. C-H stretching ethyl-bridge
66	3189.7	2849.9	0.91	0.00	3067		=	asym. C-H stretching
67	3190.2	2850.3	1.49	0.92			=	asym. C-H stretching
68	3192.8	2852.4	2.26	0.01			=	asym. C-H stretching

4.4. Vibrational analysis of [Mo(CO)3(TPn)] (4)

69	3205.0	2862.4	0.98	0.00			=	asym. C-H stretching
70	3205.3	2862.6	7.11	1.58			/	sym. C-H stretching
71	3211.1	2867.4	0.009	0.00			=	asym. C-H stretching
72	3215.6	2871.1	0.82	0.02			/	sym. C-H stretching
73	3220.6	2875.1	0.50	0.04			=	asym. C-H stretching
74	3220.7	2875.2	0.60	0.59			=	asym. C-H stretching

⊥ : TDM is perpendicular to the surface oriented

/ : TDM is skew to the surface oriented

= : TDM is parallel to the surface

5. X-Ray Photoelectron Spectroscopy (XPS)

5.1. XPS measurements of [Mo(CO)$_3$(TPn)] (4) on Au(111)

5.1.1. Monolayer on Au(111)

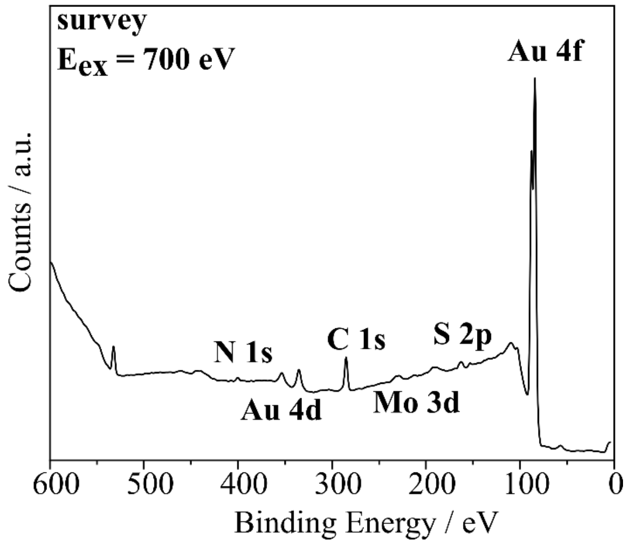

Figure S16: Survey spectrum of [Mo(CO)$_3$(TPn)] (**4**) as a monolayer on Au(111).

Table S3: Fitting parameters of the XP spectra of [Mo(CO)$_3$(TPn)] (**4**) as a monolayer on Au(111).

Component	Signal	Binding Energy / eV	Area / %	fwhm
C-C	C 1s	284.7	60	1.1
C-N/S	C 1s	285.2	32	1.1
C-O	C 1s	286.3	8	1.1
pyridine	N 1s	398.8	100	1.5
Mo(0)	Mo 3d	227.6 / 230.7	21 / 14	1.0
Mo(IV)	Mo 3d	229.1 / 232.2	24 / 15	1.1
Mo(VI)	Mo 3d	231.9 / 235.0	16 / 10	1.1
S-different thiolate	S 2p	161.2 / 162.4	22 / 11	0.8
S-Au	S 2p	162.0 / 163.2	15 / 7	1.1
S-thioether	S 2p	164.1 / 165.3	30 / 15	1.1

5.1.2. Thicklayer on Au(111)

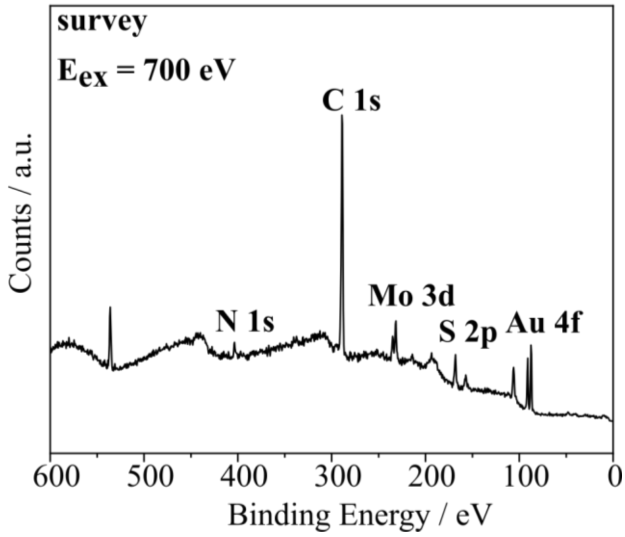

Figure S17: Survey spectrum of [Mo(CO)$_3$(TPn)] (**4**) as a thicklayer on Au(111).

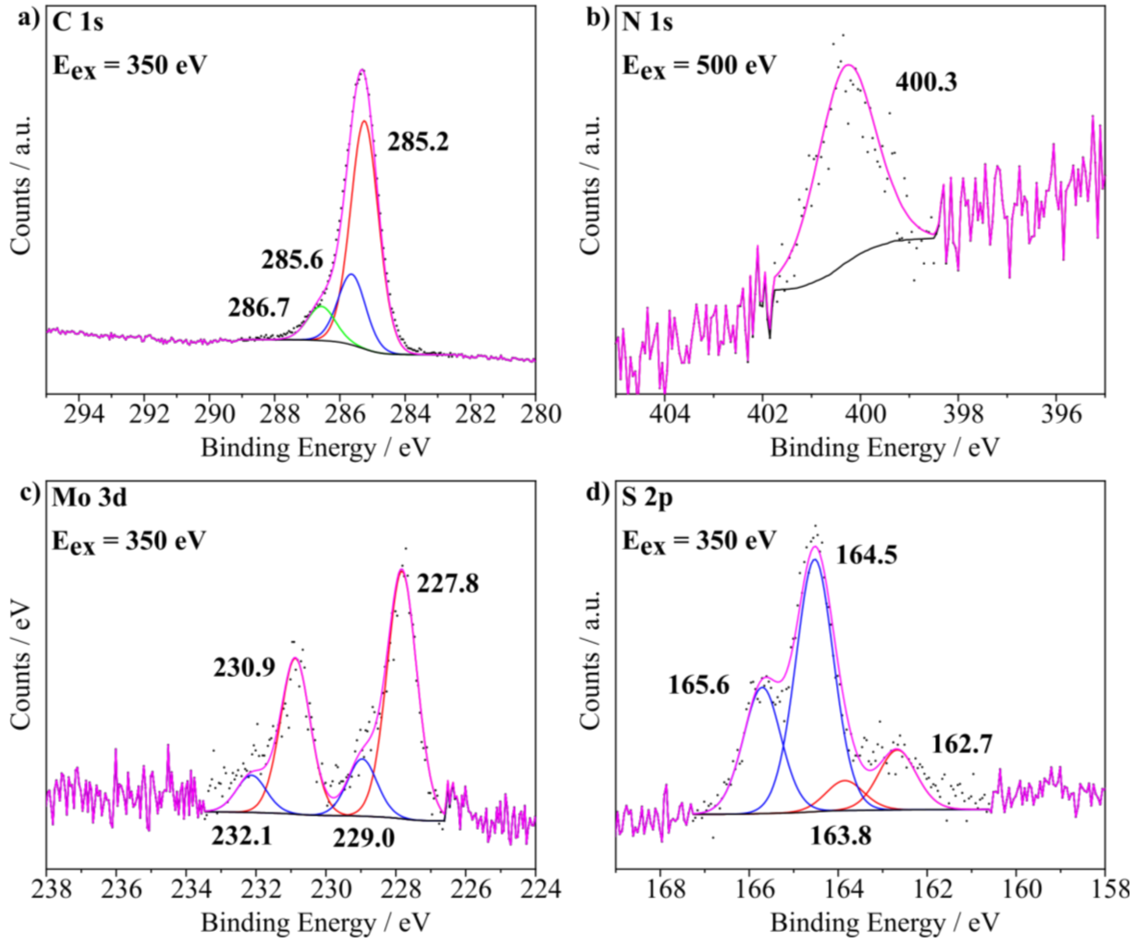

Figure S18: C 1s (a), N 1s (b), Mo 3d (c) and S 2p (d) spectra of [Mo(CO)$_3$(TPn)] (**4**) as a thicklayer on Au(111).

5.2. XPS measurements of dithia-2.1.1-(2,6)-pyridinophane (3) on Au(111)

Table S4: Fitting parameters of the XP spectra of [Mo(CO)$_3$(TPn)] (**4**) as a thicklayer on Au(111).

Component	Signal	Binding Energy / eV	Area / %	fwhm
C-C	C 1s	285.2	69	1.0
C-N/S	C 1s	285.6	21	1.0
C-O	C 1s	286.7	10	1.0
pyridine	N 1s	400.3	100	1.4
Mo(0)	Mo 3d	227.8 / 230.9	50 / 31	1.0
Mo(IV)	Mo 3d	229.0 / 232.1	12 / 7	1.0
S-Au	S 2p	162.7 / 163.8	13 / 6	1.0
S-thioether	S 2p	164.5 / 165.6	54 / 27	1.0

5.2. XPS measurements of dithia-2.1.1-(2,6)-pyridinophane (3) on Au(111)

5.2.1. Monolayer on Au(111)

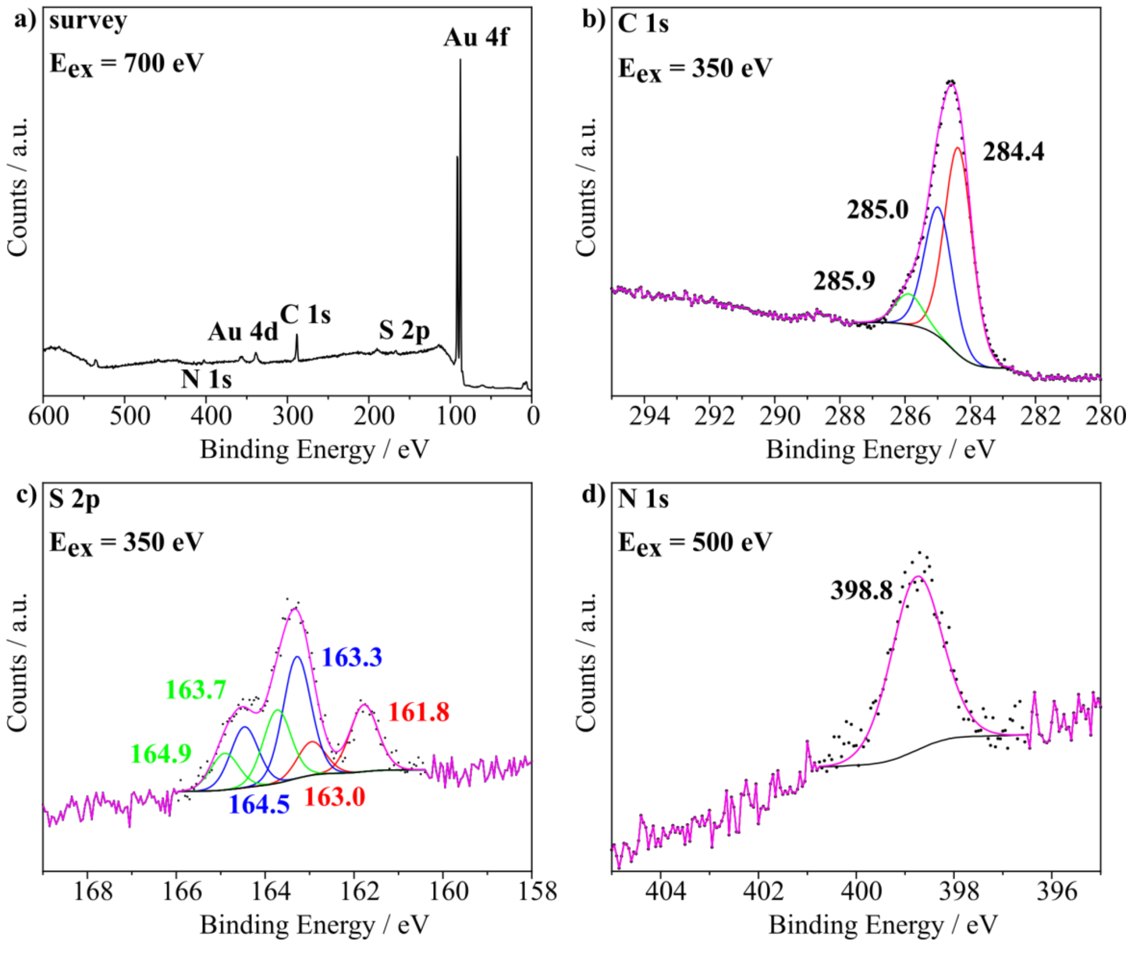

Figure S19: Survey spectrum (a), C 1s (b), S 2p (c) and N 1s (d) spectra of dithia-2.1.1-(2,6)-pyridinophane (**3**) as a monolayer on Au(111).

5.2.2. Thicklayer on Au(111)

Table S5: Fitting parameters of the XP spectra of dithia-2.1.1-(2,6)-pyridinophane (**3**) as a monolayer on Au(111).

Component	Signal	Binding Energy / eV	Area / %	fwhm
C-C	C 1s	285.2	56	1.0
C-N/S	C 1s	285.6	35	1.0
C-ox.	C 1s	285.9	9	1.0
pyridine	N 1s	398.8	100	1.2
S- different thiolate	S 2p	161.8 / 163.0	17 / 8	0.7
S-Au	S 2p	163.3 / 164.5	31 / 16	0.8
S-thioether	S 2p	163.7 / 164.9	19 / 9	0.8

5.2.2. Thicklayer on Au(111)

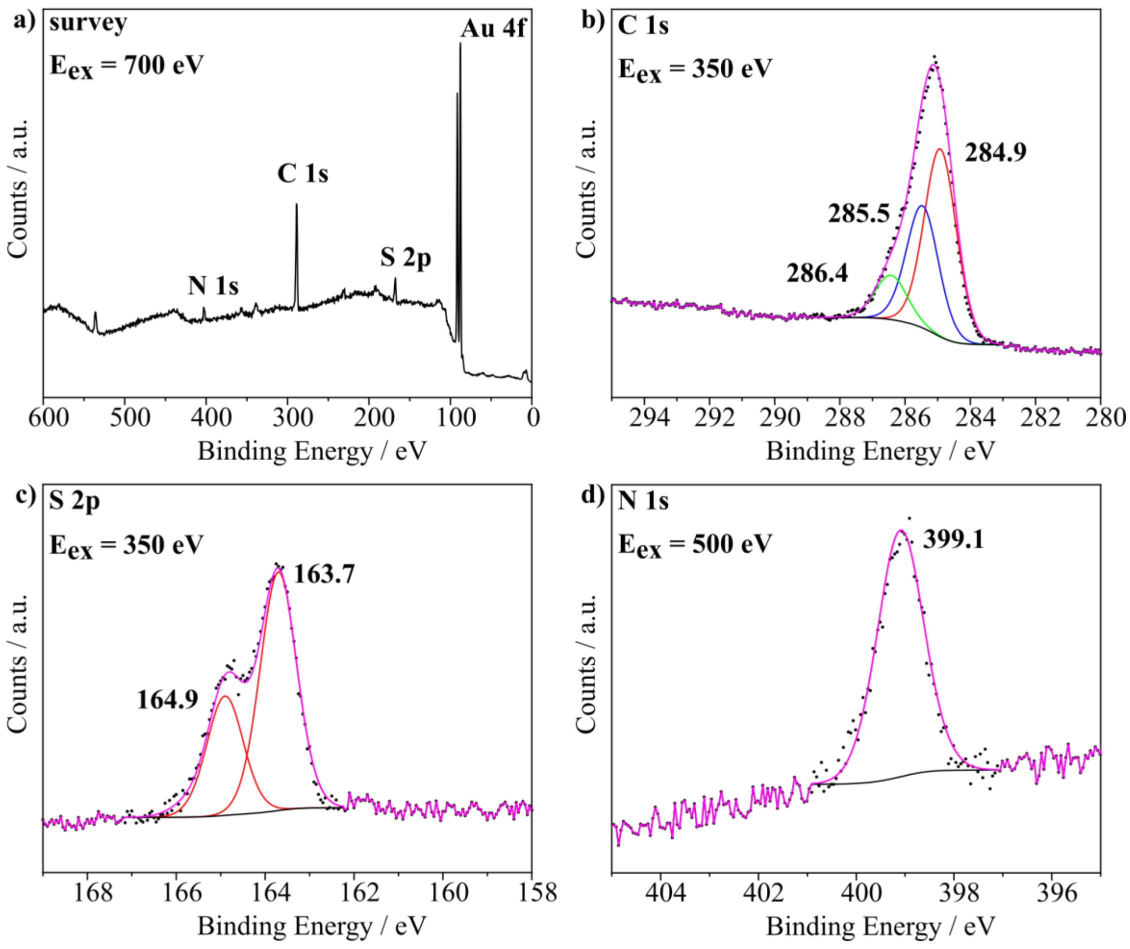

Figure S20: Survey spectrum (a), C 1s (b), S 2p (c) and N 1s (d) spectra of dithia-2.1.1-(2,6)-pyridinophane (**3**) as a thicklayer on Au(111).

5.2.2. Thicklayer on Au(111)

Table S6: Fitting parameters of the XP spectra of dithia-2.1.1-(2,6)-pyridinophane (**3**) as a thicklayer on Au(111).

**Component**	**Signal**	**Binding Energy / eV**	**Area / %**	**fwhm**
C-C	C 1s	284.9	53	1.2
C-N/S	C 1s	285.5	35	1.2
C-ox.	C 1s	286.4	12	1.2
pyridine	N 1s	399.1	100	1.1
S-thioether	S 2p	163.7 / 164.9	62 / 31	1

6. Reactivity toward oxygen (O_2)

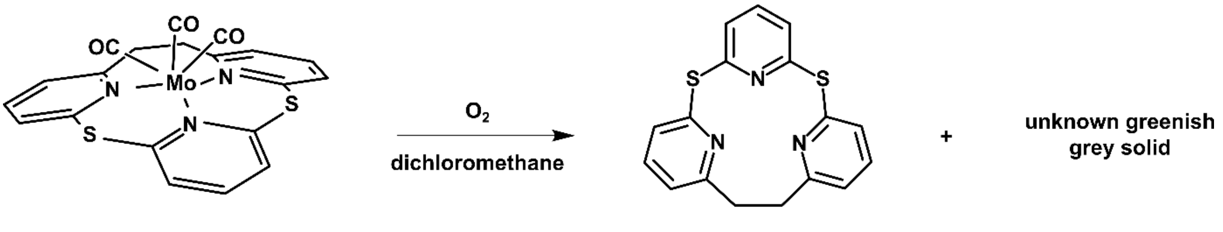

4 3

Scheme S1: The reaction of [$Mo(CO)_3$(TPn)] (**4**) with oxygen (O_2) in dichloromethane results in ligand **3** and an unknown greenish grey solid.

Under inert atmosphere, [$Mo(CO)_3$(TPn)] (**4**) (50 mg, 99 µmol) was dissolved in 10 mL dry dichloromethane. The inert atmosphere was replaced by a 100% oxygen atmosphere. The red solution was stirred at for 7 days at room temperature. Over the course of this period, the solution gradually turned darker, ultimately yielding a dark green suspension by the end. The suspension was then filtered, washed with 10 mL of dichloromethane and dried under vacuum. The solvent from the filtrate was removed by vacuum, resulting a white solid.

Yield: 8 mg of greenish gray solid and 20 mg of white solid (filtrate).

The white solid was investigated by ^{1}H-NMR (Figure S20). The greenish gray solid was investigated by Powder X-ray diffraction (XRPD) and compared to molybdenum(IV)oxide (MoO_2)[1] and molybdenum(VI)oxide (MoO_3)[2] (Figure S21). Furthermore, the gray solid was compared to the IR spectra of complex **4** and ligand **3** (Figure S22).

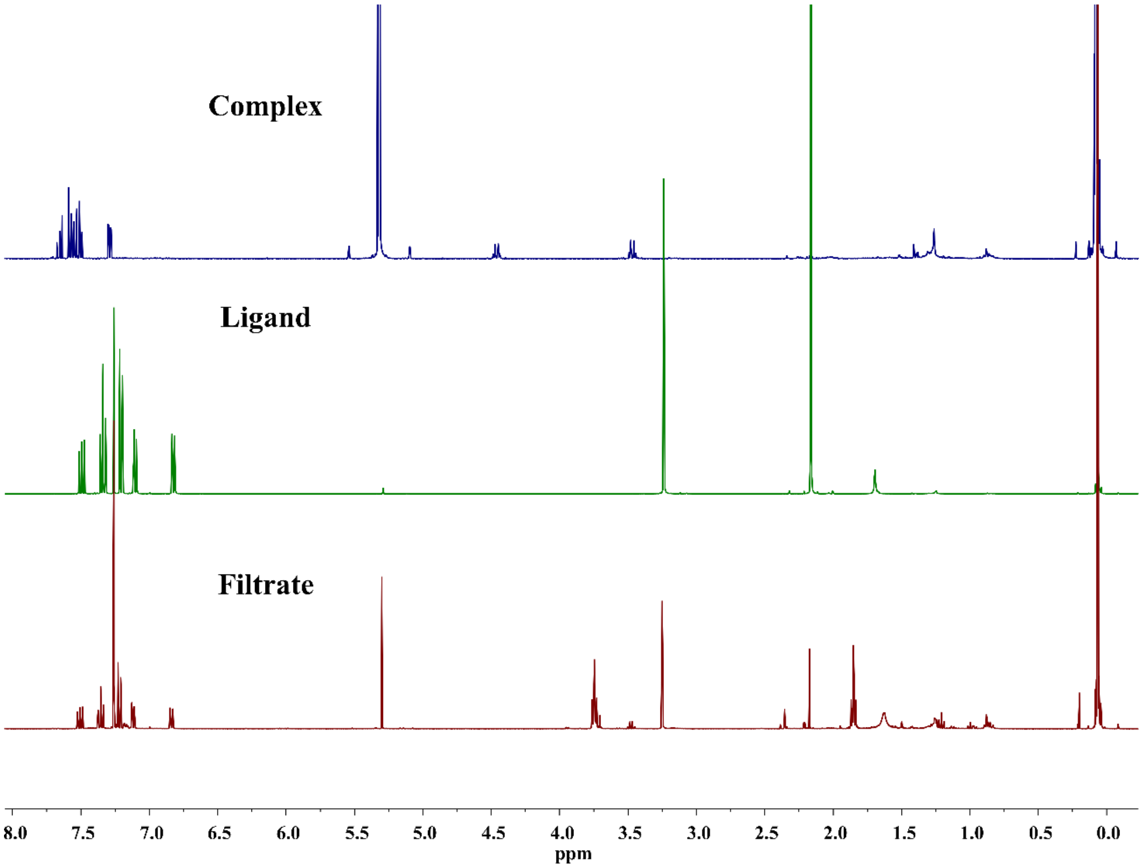

Figure S21: ^{1}H-NMR of the filtrate compared to the spectra of complex **4** and ligand **3**. Some impurities can be seen but generally the signals fit perfectly to the ones of the ligand **3**.

Reactivity toward oxygen (O2)

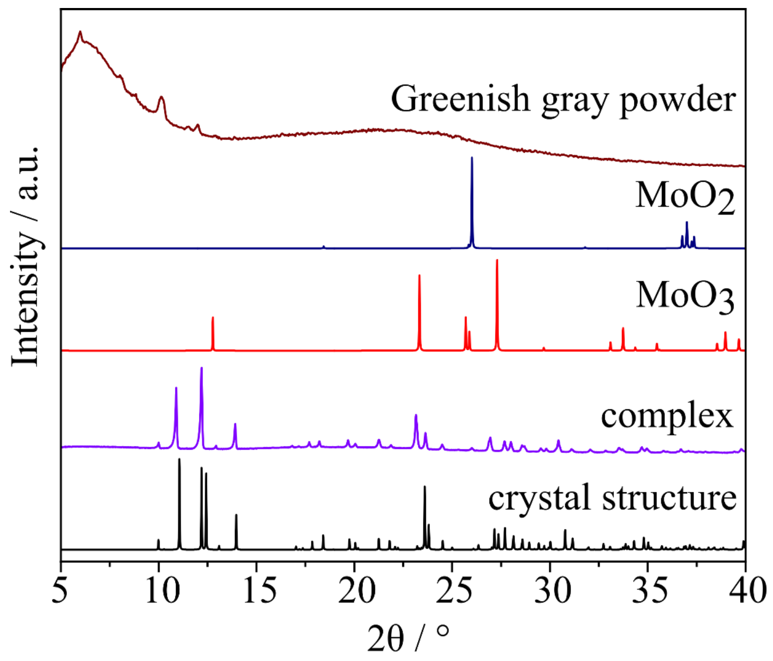

Figure S22: XRD pattern spectrum from 5° to 40°of the crystal structure of $[Mo(CO)_3(TPn)]$ (**4**) (black), the synthesized complex **4** (purple), the calculated spectra of MoO_2 (red) and MoO_3 (blue), and the unknown residue (brown).

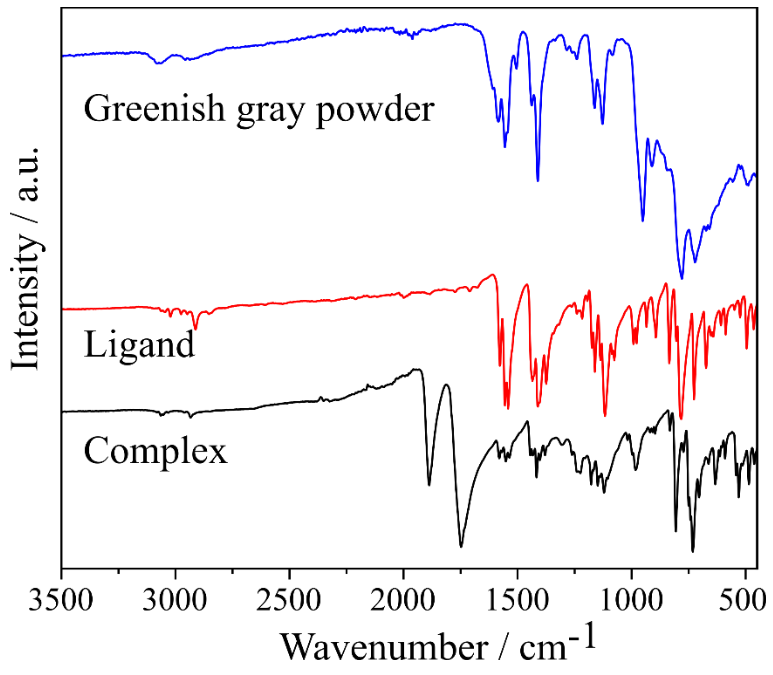

Figure S23: IR spectra of complex **4** (black), ligand **3** (red) in comparison to the IR spectrum of the gray powdered residue (blue).

7. Computational Details

7.1. Coordinates of DFT-calculated structures of [Mo(CO)$_3$(TPn)] (4)

Table S7: Atomic coordinates of the DFT-calculated structure of [Mo(CO)$_3$(TPn)] (**4**).

	x	y	z		x	y	z
C	0.61353	3.57182	-1.43574	C	-0.92304	-3.26795	0.711924
C	1.97837	3.56123	-1.72047	H	-3.09097	-3.22657	0.60700
C	2.70397	2.39632	-1.47524	H	-2.40628	-2.05843	1.74339
C	2.05304	1.31881	-0.86667	Mo	0.030350	-0.00494	1.28085
C	0.04770	2.44598	-0.82933	H	-0.01929	4.42293	-1.69034
N	0.76826	1.36794	-0.46200	H	2.45772	4.42872	-2.17971
S	2.99709	-0.15309	-0.57735	H	3.75229	2.30228	-1.76117
S	-1.69201	2.48389	-0.49105	H	2.94991	-1.94462	-2.7183
C	-2.19961	0.81285	-0.87311	H	1.54379	-3.99272	-3.19313
C	1.82008	-1.44426	-0.95535	H	-0.26513	-4.57718	-1.54637
N	0.78679	-1.69694	-0.12159	H	-3.81839	-2.58674	-1.45521
C	2.11832	-2.22890	-2.07357	H	-3.27629	1.54382	-2.58873
C	1.34470	-3.35915	-2.32597	H	-4.31744	-0.71292	-3.05700
C	0.08376	-2.84323	-0.32202	H	-1.12854	-4.33527	0.54614
C	0.33352	-3.67326	-1.42166	H	-0.43902	-3.18174	1.69357
C	-3.06577	0.67690	-1.96239	C	-0.61908	1.54874	2.29997
N	-1.85205	-0.21340	-0.06482	C	1.72328	0.23185	2.25495
C	-2.47381	-1.40616	-0.26166	C	-0.513292	-1.05345	2.82869
C	-3.64107	-0.56685	-2.21172	O	-0.95532	2.45559	2.94563
C	-3.35244	-1.607540	-1.33291	O	2.68995	0.40367	2.87810
C	-2.27521	-2.50296	0.748182	O	-0.81767	-1.65879	3.78303

7.2. Coordinates of DFT-calculated structures of [MoO_3(TPn)] (5)

Table S8: Atomic coordinates of the DFT-calculated structure of [MoO_3(TPn)] (**5**).

	x	y	z		x	y	z
C	2.03697	3.34217	-2.13168				
C	0.69126	3.45125	-1.80404	C	-1.89477	-2.23760	-1.42028
C	2.72857	2.20288	-1.73751	C	-1.15931	-3.06220	-0.33520
C	2.05986	1.23464	-0.97237	H	-1.49568	-2.75306	0.66692
C	0.08698	2.44167	-1.03834	H	-1.40389	-4.12531	-0.46626
N	0.77240	1.36354	-0.59206	H	-1.18537	-1.94596	-2.21103
S	3.12877	-0.07713	-0.45353	H	-2.64797	-2.88333	-1.89514
S	-1.59512	2.81813	-0.63818	H	0.09076	4.29870	-2.13710
C	-2.48793	1.29010	-0.59488	H	2.53067	4.11827	-2.72092
C	2.23594	-1.60895	-0.45771	H	3.76994	2.04113	-2.01737
C	-3.87917	1.40604	-0.43959	H	0.62455	-5.05985	-0.53450
N	-1.86522	0.12796	-0.77529	H	4.15571	-2.58274	-0.70755
C	-2.60412	-0.98598	-0.96797	H	3.12659	-4.87921	-0.67324
C	-4.63295	0.24414	-0.53293	H	-4.33992	2.37956	-0.26338
C	-3.99420	-0.96275	-0.83730	H	-5.71783	0.28056	-0.40583
N	0.90903	-1.70123	-0.31218	H	-4.56911	-1.87893	-0.98220
C	3.07967	-2.72338	-0.59280	Mo	-0.06522	0.05319	1.23130
C	2.50436	-3.98537	-0.58569	O	-0.80368	1.50676	1.76650
C	0.33707	-2.93164	-0.37947	O	1.47117	-0.02340	1.98845
C	1.11633	-4.08677	-0.50301	O	-1.03832	-1.23609	1.83854

7.3. Calculation of IRRA spectrum of [Mo(CO)$_3$(TPn)] (4)

The frequency calculation of the IRRA spectrum was performed as a single molecule calculation (gas phase), in which the vibrational modes in the z direction were reinforced and in the xy plane were diminished. Only the component along the z-axis is considered due to the surface selection rule of IRRA spectroscopy, because the metal surface lies in the xy plane.
In Table S9 a selection of vibrational modes from the vibrational analysis (Table S2) are shown of which the values of the transition dipole moments along the three axes are displayed.

Table S9: Selection of vibrational modes from the vibrational analysis (Table S2) with values of the transition dipole moments (TDM) along the three axes.

**Mode**	**Calc. freq. uncorr.**	**Calc. freq. corr.**	**Sum of intensities**	**TDM$_x$**	**TDM$_y$**	**TDM$_z$**	**Intensity IRRAS**
53	1590.2	1540.4	17.62	0.01	-4.20	-0.01	0.00
54	1599.9	1548.5	22.27	4.28	0.02	1.96	3.83
55	1603.3	1551.2	6.41	0.03	-2.51	0.01	0.00
56	1614.7	1560.5	3.39	1.38	0.00	1.19	1.42
57	1629.3	1572.4	1.14	0.01	1.03	0.00	0.00
58	1634.0	1576.3	16.69	4.01	0.00	0.79	0.63
59	1938.5	1825.5	1051.93	31.19	0.07	-8.33	69.46
60	1975.8	1856.1	892.50	0.04	-30.11	-0.07	0.00
61	2035.1	1904.7	1906.01	-4.62	0.11	-43.42	1884.96

8. Literature

[1] G. Andersson, A. Magneli, L. G. Sillén, M. Rottenberg, *Acta Chem. Scand* **1950**, *4*, 793.
[2] T. Leisegang, A. A. Levin, J. Walter, D. C. Meyer, *Cryst. Res. Technol.* **2005**, *40*, 95.
